# Exosomes from CD99-deprived Ewing sarcoma cells reverse tumor malignancy by inhibiting cell migration and promoting neural differentiation

**DOI:** 10.1038/s41419-019-1675-1

**Published:** 2019-06-17

**Authors:** Alessandra De Feo, Marika Sciandra, Manuela Ferracin, Federica Felicetti, Annalisa Astolfi, Ymera Pignochino, Piero Picci, Alessandra Carè, Katia Scotlandi

**Affiliations:** 1 Laboratory of Experimental Oncology, IRCCS Istituto Ortopedico Rizzoli, Via Di Barbiano 1/10, 40136 Bologna, Italy; 2 Department of Experimental, Diagnostic and Specialty Medicine, DIMES, University of Bologna, Bologna, Italy; 3 Department of Oncology and Molecular Medicine, Istituto Superiore di Sanità, Rome, Italy; 4 “Giorgio Prodi” Cancer Research Center, University of Bologna, Bologna, Italy; 5 Sarcoma Unit, Medical Oncology, Candiolo Cancer Institute, FPO, IRCCS, Candiolo, Turin Italy; 6 Oncology Unit, Center for Gender Medicine, Istituto Superiore di Sanità, Rome, Italy

**Keywords:** Paediatric cancer, Sarcoma, miRNAs

## Abstract

Ewing sarcoma (EWS) is an aggressive mesenchymal tumor with unmet clinical need and significant social impacts on children, adolescents, and young adults. CD99, a hallmark surface molecule of EWS, participates in crucial biological processes including cell migration, differentiation, and death. EWS cells can release CD99 through exosomes (EXOs), specialized extracellular vesicles with major cell communication roles. Here we show that, as a consequence of CD99 silencing, EWS cells deliver exosomes with oncosuppressive functions that significantly reduce tumor aggressiveness. These CD99-lacking microvesicles modulate gene expression of the EWS-recipient cells, reduce proliferation and migration, in turn inducing a more-differentiated less-malignant phenotype. The most relevant effects were detected on the activator protein-1 signaling pathway whose regulation was found to be dependent on the specific cargo loaded in vesicles after CD99 shutdown. Investigation of the miRNA content of CD99-deprived EXOs identified miR-199a-3p as a key driver able to reverse EWS malignancy in experimental models as well as in clinical specimens. All together, our data provide evidence that the abrogation of CD99 in EWS tumor cells leads to produce and release EXOs capable to transfer their antineoplastic effects into the nearby tumor cells, suggesting a novel atypical role for these microvesicles in reversion of malignancy rather than in priming the soil for progression and metastatic seeding. This conceptually innovative approach might offer a new therapeutic opportunity to treat a tumor still refractory to most treatments.

## Introduction

Ewing sarcoma (EWS) is a highly aggressive mesenchymal tumor affecting children, adolescents, and young adults. Genetically, three major sequencing studies have shown that EWS is characterized by a striking paucity of somatic mutations^1–3^.

Apart from an oncogenic hybrid transcript that derived from the fusion of the EWSR1 gene with a member of the E26 transformation-specific (ETS) transcription factor family, the other most common alterations are found in STAG2 (15–20% of cases), CDKN2A (12% of cases), and TP53 (6–10% of cases). The quiet genome of EWS, together with the high levels of inter- and intratumoral heterogeneity^4^, has prevented the identification and prioritization of preclinical candidates that might improve patient outcomes. Even the use of immune checkpoint inhibitors, which has been reported to be successful in several tumor types^5–7^, has produced discouraging results in EWS^8^. Thus, very few advances in the treatment of EWS have been recorded in the last decade, and current therapy remains confined to conventional chemotherapy. Survival of patients with metastases at diagnosis remains dismal^9,10^, and very few treatment options can be offered to the patients with localized tumors that recur after first-line treatments. In addition, even in more favorable situations in which patients with localized tumors at diagnosis respond well to therapy (survival rate up to 70–75% at 5 years), the patients experience significant side effects that limit their quality of life. Therefore, there is a strong demand from patients, families, and physicians for additional therapies. It is very likely that novel therapeutic approaches to combat this very aggressive and elusive tumor may be derived only from the development of innovative strategies exploiting new biomolecular features of the disease. The latest advances have been derived from cutting-edge technological applications. However, it is necessary to return to biology to properly contextualize this knowledge. In this paper, we focused on exosomes (EXOs), a subset of extracellular vesicles recognized to have a major role in cellular communications^11^. EXOs carry and transfer many types of cargo, including proteins, mRNAs and miRNAs, which are major components of the mechanisms used by tumor-derived EXOs to carry out their functions. Indeed, EXOs can be absorbed by recipient cells, thereby transferring both signaling proteins and genetic information and exerting observable and function-altering effects. We have recently demonstrated that CD99, a cell surface molecule that is a hallmark of EWS^12^, can be released by EWS cells, mainly through EXOs^13^. CD99 is involved in crucial biological processes, including cell migration, cell death, and differentiation^14^. EWS cells harboring the oncogenic EWS-ETS fusion gene are inhibited with regards to growth, migration and metastasis and are prone to differentiate toward neural lineage if deprived of CD99^12^. In this study, we thoroughly investigate the capability of EXOs lacking CD99 (CD99neg EXOs), released from CD99-silenced EWS cells, to interfere with the aggressive profile of tumor cells. Exploring the specific cargo loaded in these vesicles, we focused on miRNA content to identify players able to affect malignancy and to shape the genetic landscape of EWS neoplastic cells.

## Results

### CD99neg EXOs halt EWS aggressiveness and shape neural differentiation

**Fig. 1:**
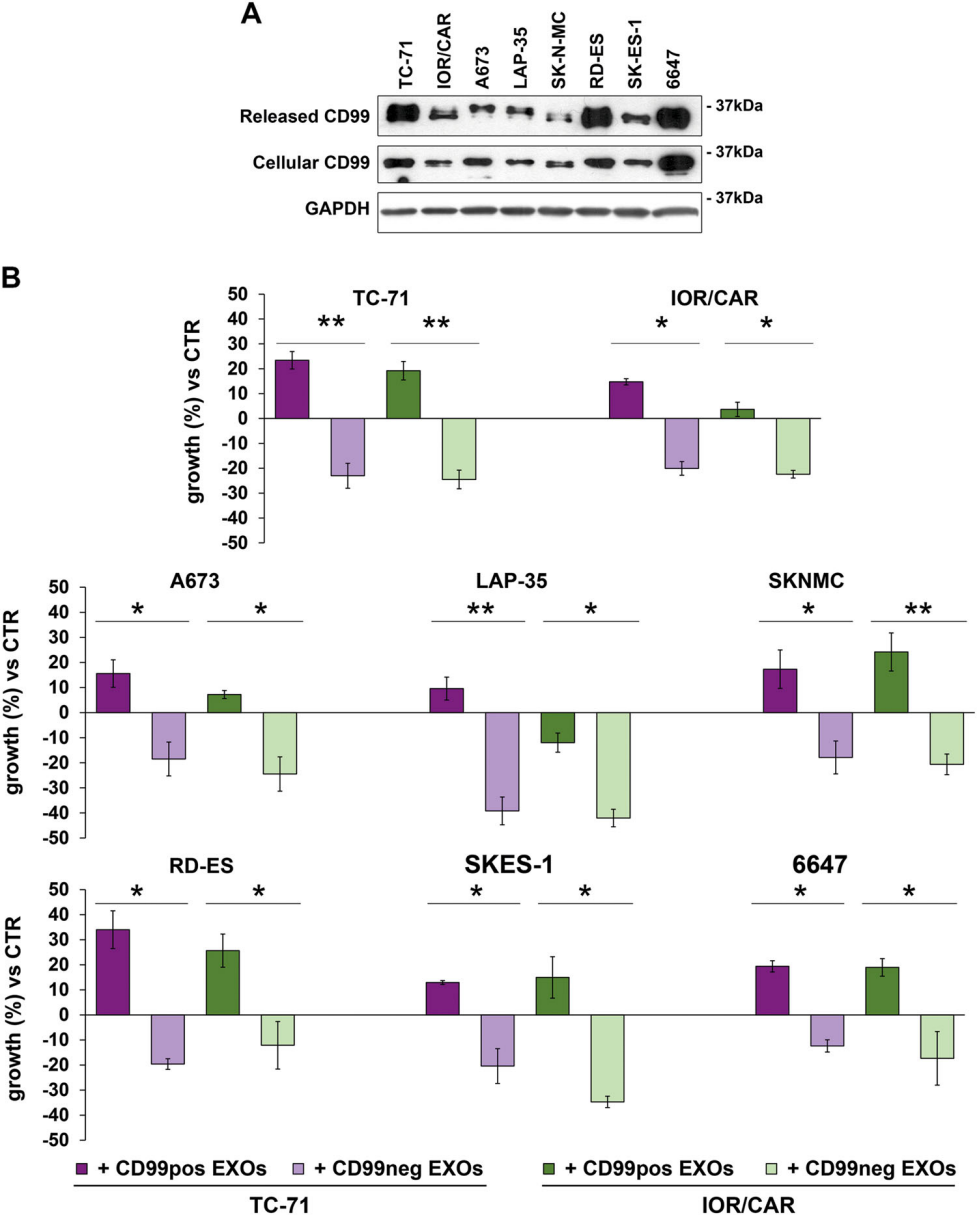
Released CD99 affects cell growth in EWS recipient cells. **a** Representative western blotting showing CD99 secreted in the supernatants of eight EWS cell lines; CD99 protein expression in the corresponding EWS cells is shown for comparison. GAPDH was used as a loading control. **b** Evaluation of cell growth in serum-free condition in EWS cells receiving CD99pos- or CD99neg EXOs isolated from the TC-71 (purple bars) and IOR/CAR (green bars) experimental models. Columns show the mean values of at least two independent biological experiments with two replicates/each, and the bars represent the SE (*p < 0.05, **p < 0.01, Student's *t-*test, *n* = 2–4)

**Fig. 2:**
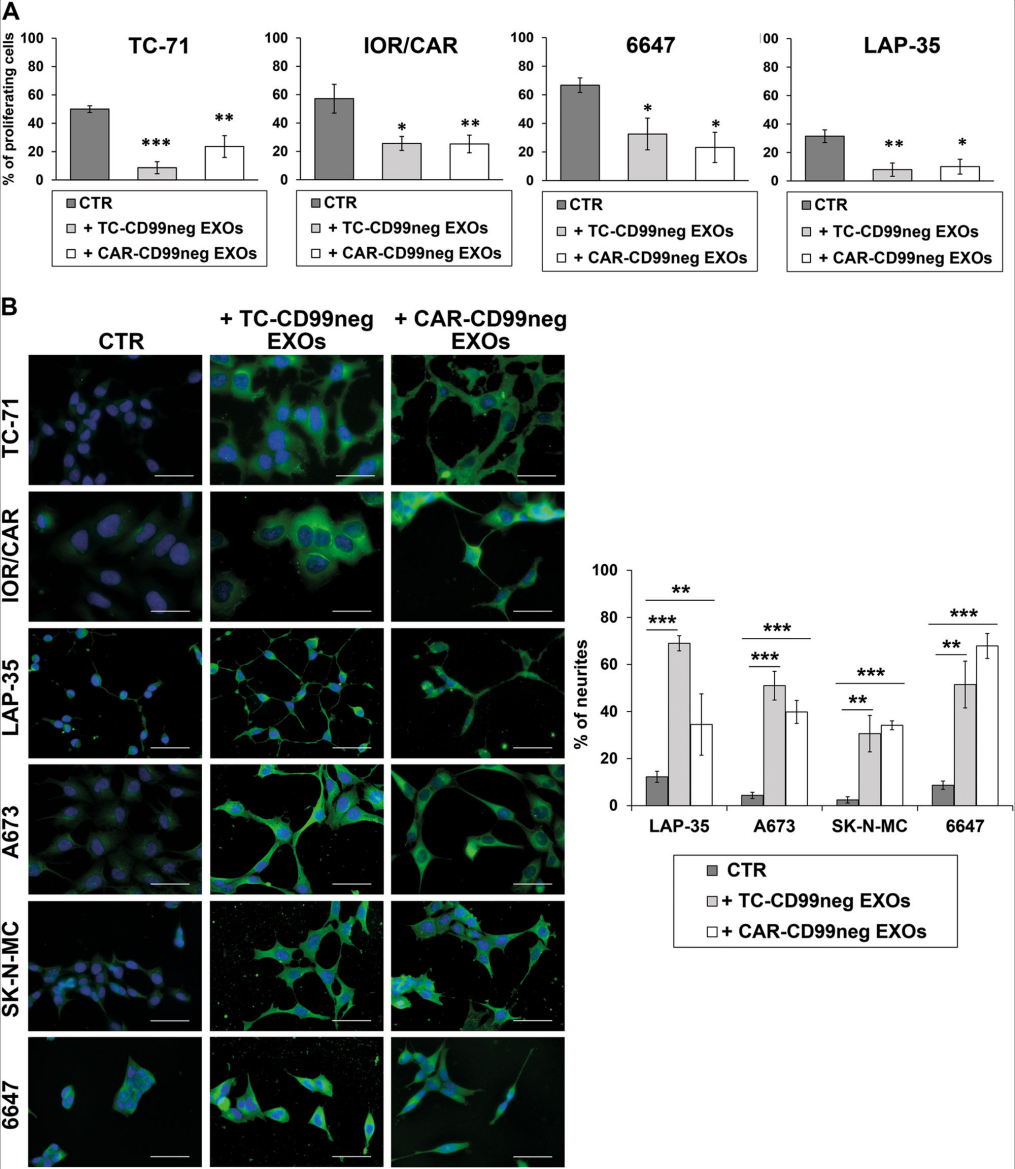
CD99neg EXOs reduce the proliferation rate and foster neural differentiation in EWS cells. **a** Ki-67 immunostaining was performed in a panel of EWS cell lines receiving or not receiving (CTR) CD99neg EXOs obtained from CD99-silenced cells. Ki-67-positive cells were counted, and the percentages are shown. Values are from one experiment representative of two independent biological experiments, with 6–10 fields/each. Columns show the mean values and the bars represent the SE (*p < 0.05, **p < 0.01,***p < 0.001, one-way ANOVA, *n* = 6–10). **b** Immunofluorescence staining of β-III Tubulin (green) in EWS cells receiving or not receiving (CTR) CD99neg EXOs isolated from TC-CD99-shRNA or CAR-CD99-shRNA cells. The nuclei were labeled by Hoechst 33258 (blue). Representative images are displayed. Scale bars: 50 µm. Quantitative analysis of neurite outgrowth (%) in EWS cells treated or not treated (−) with TC- or CAR-CD99neg EXOs from CD99-silenced models. Values are from one experiment representative of two independent biological experiments, with 6–10 fields/each. Columns show the mean values and the bars represent the SE (**p < 0.01, ***p < 0.001, one-way ANOVA, *n* = 6–10)

**Fig. 3:**
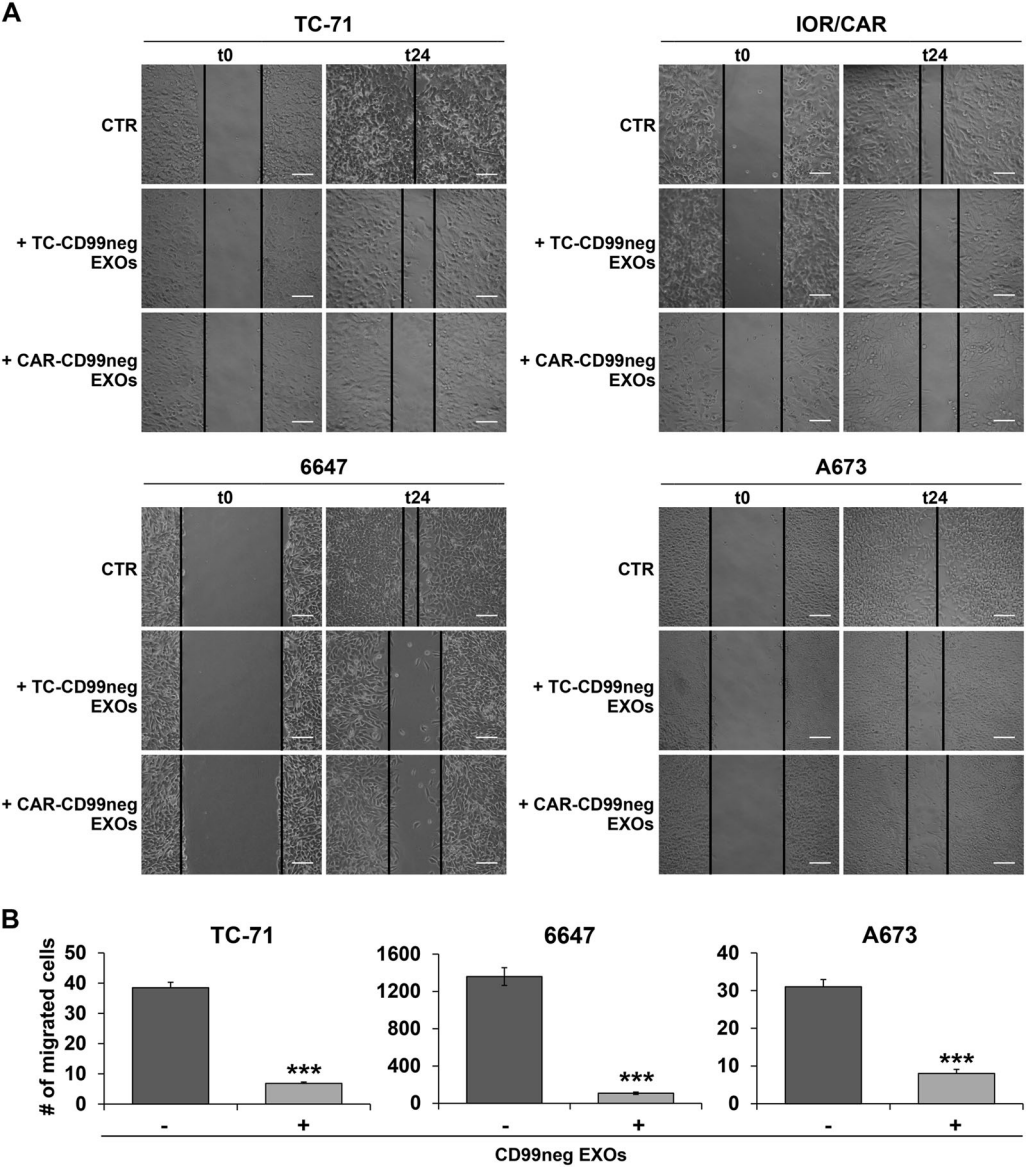
CD99neg EXOs promote reductions in cell migration. **a** Wound-healing in EWS cells receiving or not receiving (CTR) CD99neg EXOs purified from CD99-silenced TC-71 or IOR/CAR cell lines. The assay was performed over 24 h. Scale bars: 100 µm. **b** Motility assay with transwell chambers in EWS cell lines untreated (−) or pretreated for 30 min with CD99neg EXOs. The data are shown as the mean ± SE of two independent biological experiments with three replicates/each (***p < 0.001, Student's *t*-test, *n* = 6)

In a panel of eight patient-derived EWS cell lines, we evaluated the levels of released CD99 to confirm observations obtained in genetically modified cells^13^. Indeed, all the EWS cells released CD99, and its levels in cell supernatants were consistent with the cellular expression of the molecule (Fig. 1a) and were positively correlated with tumor cell aggressiveness (Table 1). Released CD99 was demonstrated to be mostly associated with EXOs^13^. Therefore, we used two EWS cell lines stably deprived of CD99, the TC-CD99-shRNA and CAR-CD99-shRNA variants^12,13^, as donor cells of CD99neg EXOs (Supplementary Fig. 1a) and verified their impact on EWS malignancy. The EXOs were purified with the ExoQuick-TC (EQ) system from conditioned media of CD99-lacking cells, and the sizes and amounts of EXOs characterized by using NanoSight technology (Supplementary Fig. 1b). CD99 expression on the EXO surface was determined by flow cytometry (Supplementary Fig. 1c). CD99neg EXOs were then incubated with EWS cell lines. The internalization of the EXOs into acceptor cells was verified by staining the cells with the fluorescent fatty acid molecule BODIPY® FL C16, which, as it is incorporated into membrane phospholipids, made possible to label and visualize the EXOs^15^. A similar uptake by target cells of CD99neg EXOs or EXOs isolated from CD99-expressing parental cells (CD99pos EXOs) was confirmed (Supplementary Fig. 2a, b). EWS cells exposed to and incorporating CD99neg EXOs displayed lower cell growth than cells absorbing CD99pos EXOs (Fig. 1b). There was a reduction in cell proliferation, as shown by Ki-67-labeling index (Fig. 2a), but no significant induction of cell death (Supplementary Fig. 3a, b). In addition, EWS cells receiving CD99neg EXOs displayed increased neural differentiation, as shown by the expression of β-III Tubulin and the number of neurites (Fig. 2b). Thus, EXOs derived from CD99-silenced EWS cells are able to induce the same phenotype that we have proven to be associated with stable CD99 silencing^12,13^. Accordingly, when CD99-deprived cells, which showed a more differentiated phenotype, received CD99pos EXOs, the expression of β-III Tubulin was inhibited (Supplementary Fig. 4a). In addition, EWS cells exposed to CD99neg EXOs exhibited significantly reduced migration (Fig. 3a, b), whereas CD99-silenced cells, which showed a reduced migratory capability with respect to controls, regained their ability when exposed to CD99pos EXOs derived from parental EWS cells (Supplementary Fig. 4b). To exclude aspecific effects that simply reflect a cellular response to EXO exposure at high concentration, both CD99pos- or CD99neg EXOs were used to treat the respective producer cells (Supplementary Fig. 5).

**Table 1:** Relationship between released CD99 and EWS malignancy in a panel of EWS cell lines

Cell line	OD CD99^a^	Released CD99 (score)	Motility	Growth in soft agar	Bone metastases	
					Incidence (%)	Latency (days)
			*r* = 0.92	*r* = 0.95	*r* = 0.96	*r* = −0.46
			*P* = 0.027	*P* = 0.013	*P* = 0.011	*P* = −0.437
6647	27,812	High	487 ± 44	1259 ± 99	9/9 (100%)	33 ± 1
TC-71	20,199	High	148 ± 13	1048 ± 28	23/29 (79%)	44 ± 5
IOR/CAR	10,304	Medium	22 ± 5	422 ± 70	2/10 (20%)	109 ± 5
LAP-35	8441	Medium–low	23 ± 3	5 ± 2	0/5 (0%)	ND
SK-N-MC	5898	Low	42 ± 13	256 ± 21	1/5 (20%)	42

^a^ Released CD99 levels were measured by densitometric analysis after western blotting. Correlation was performed between CD99 OD and parameters of malignancy (migration, growth in anchorage-independent conditions, and capability to induce metastases). For each parameter, the correlation coefficient *r* and the significant *p* value *P* were determined by Pearson’s test (*P<0.05, Pearson’s correlation test)

In addition, CD99 was equally expressed in EWS cells receiving CD99neg EXOs and in those not receiving EXOs (Supplementary Fig. 6a, b), suggesting that the modulation of cell behavior is specific and independent of the presence of CD99 on the recipient cell surface but rather dependent on EXO cargo.

### CD99neg EXOs modulate the gene expression profile of recipient EWS cells

**Fig. 4:**
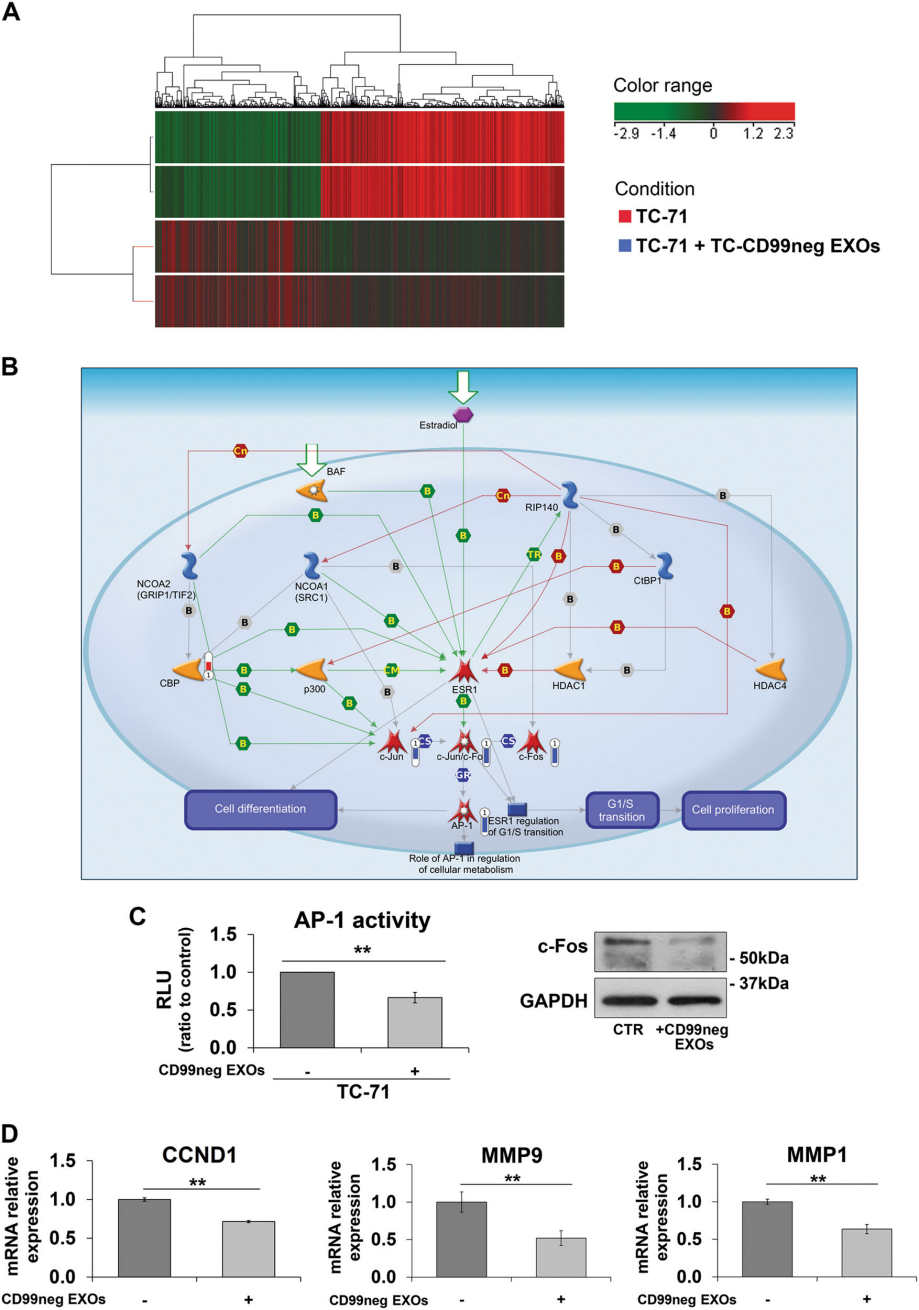
CD99neg EXOs modulate gene expression in recipient EWS cells. **a** Cluster analysis of TC-71 cells with or without CD99neg EXOs performed using a list of differentially expressed Affymetrix transcriptional clusters (adjusted *p* < 0.05). Genes (columns) and samples (rows) were grouped by hierarchical clustering (Pearson’s correlation). High expression and low expression were normalized to the average expression across all samples. **b** Top-scored map (the map with the the lowest *p* value) based on MetaCore pathway enrichment analysis of 1839 transcriptional clusters (corresponding to 508 annotated genes). Changes in gene expression after CD99neg EXOs addition are depicted on the map as a thermometer-like figures. Upregulated genes have an upward thermometer and a red color, whereas downward (blue) thermometers indicate downregulated genes in TC-71+TC-CD99neg EXOs vs TC-71 cells. **c** AP-1 transcriptional activity as assessed by luciferase assay and c-Fos expression as assessed by western blotting in TC-71 cells receiving or not receiving (CTR) CD99neg EXOs. For the luciferase assay, the data are represented as RLU and are expressed as the fold induction over the control value. The values were normalized to Renilla luciferase activity. The data are shown as the mean ± SE of three independent biological experiments, with three replicates/each (**p < 0.01, Student’s *t*-test, *n* = 6). For western blotting, GAPDH was used as a loading control. **d** The mRNA expression levels of CCND1, MMP9, and MMP1 were evaluated by qPCR in TC-71 cells treated or not treated (−) with CD99neg EXOs. The results were quantified by the 2^−ΔΔCt^ method, and untreated cells were used as calibrators (2^−ΔΔCt^ = 1). GAPDH was used as a housekeeping gene. The data are shown as the mean ± SE of three independent experiments, with two replicates/each (**p < 0.01, Student’s *t*-test, *n* = 6)

EWS cells exposed to and incorporating CD99neg EXOs showed a different gene expression profile than parental cells. Twenty-four hours after cell exposure to CD99neg EXOs, 1839 transcript clusters, including those from protein coding and non-protein coding genes, were found to be differentially expressed compared with the levels in the parental TC-71 cell line (fold-change ± 1.5; Benjamini–Hochberg-adjusted *p* value ≤ 0.05) (Fig. 4a). We performed a functional annotation of the differentially expressed genes using GeneGo MetaCore software (Thomson Reuters) and DAVID bioinformatics tool^16^. Of note, the top-scored pathway based on the enrichment analysis of GeneGo software was Development Ligand‑dependent activation of the ESR1/AP‑1 pathway (Fig. 4b, Supplementary Table 1), with significant downregulation of FOS/JUN genes (Supplementary Table 2). Consistently, EWS cells exposed to CD99neg EXOs showed significant downregulation of activator protein-1 (AP-1) activity in luciferase assays, reduced expression of c-Fos (Fig. 4c), and inhibited expression of some validated AP-1 target genes, such as CCND1, MMP9, and MMP1 (Fig. 4d).

Other major pathways in DAVID analysis of modulated genes based on KEGG pathways and Uniprot keywords are related to neural differentiation: KEGG hsa04360: Axon guidance, *p* 0.03; UP_KEYWORD Neurogenesis, *p* 0.01 (Supplementary Table 3). These results confirm that the phenotypic variations observed were at least partially due to gene expression alterations.

### miRNA cargo of CD99neg EXOs suggests miR-199a-3p as a novel tumor suppressor of EWS cells

**Fig. 5:**
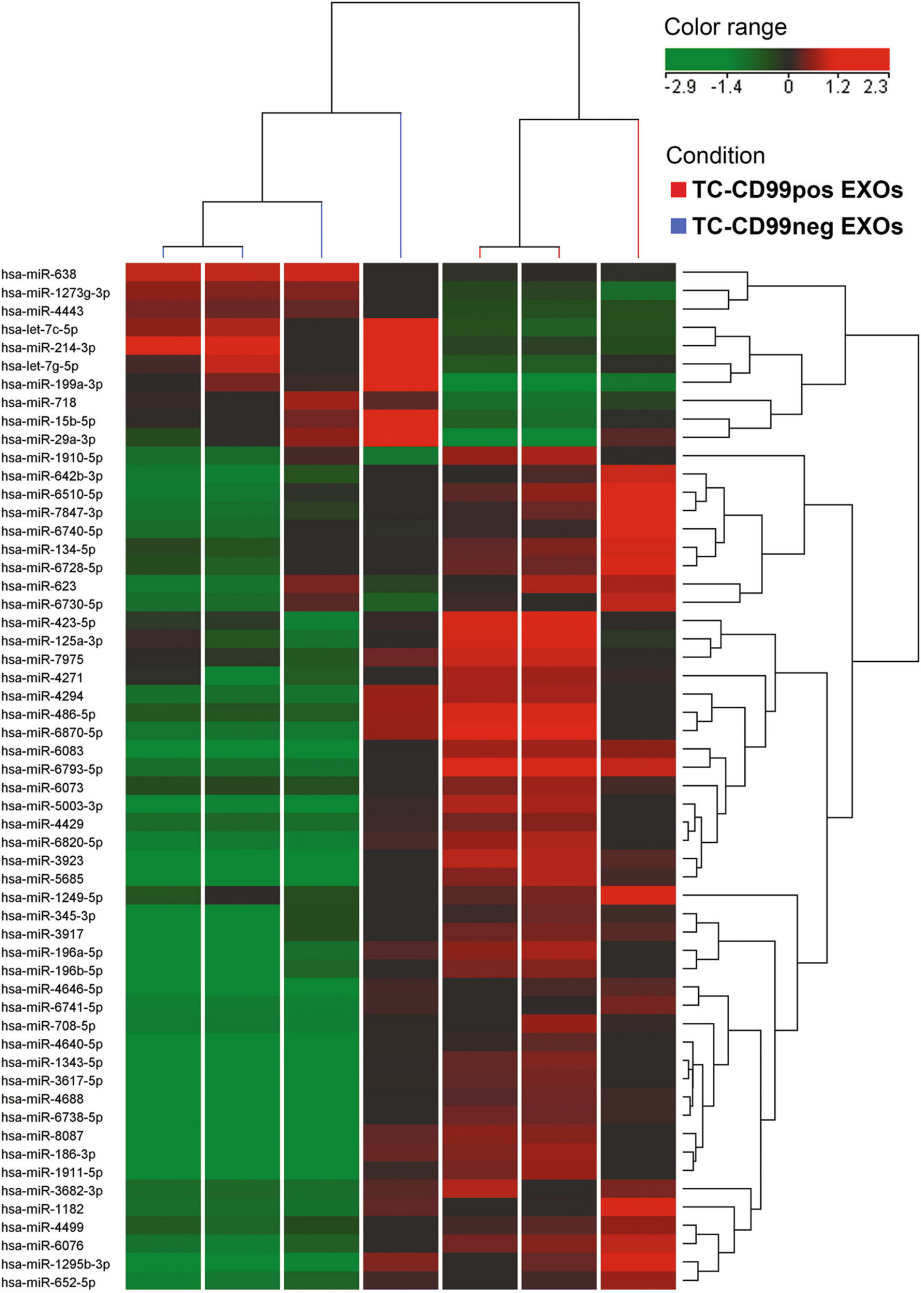
CD99pos- and CD99neg EXOs display a different miRNA cargo. Cluster analysis of TC-71 EXOs with or without CD99 performed using a list of 56 differentially expressed miRNAs (adjusted *p* < 0.2). Genes (rows) and samples (columns) were grouped by hierarchical clustering (Pearson’s correlation). High expression and low expression values were normalized to the average expression across all samples

We evaluated the miRNA content of CD99pos EXOs isolated from the parental cell line TC-71 (three samples) and of CD99neg EXOs isolated from TC-CD99-shRNA cells (four samples) by microarray analysis. We detected 350 miRNAs in the CD99neg EXOs and 398 miRNAs in the CD99pos EXOs. In addition, we identified a signature composed of 56 miRNAs (10 up- and 46 downregulated; Supplementary Table 4) that characterized the cargo of CD99neg EXOs. The results of a clustering analysis of CD99pos- and CD99neg EXOs according to the levels of these 56 miRNAs are presented in Fig. 5.

**Fig. 6:**
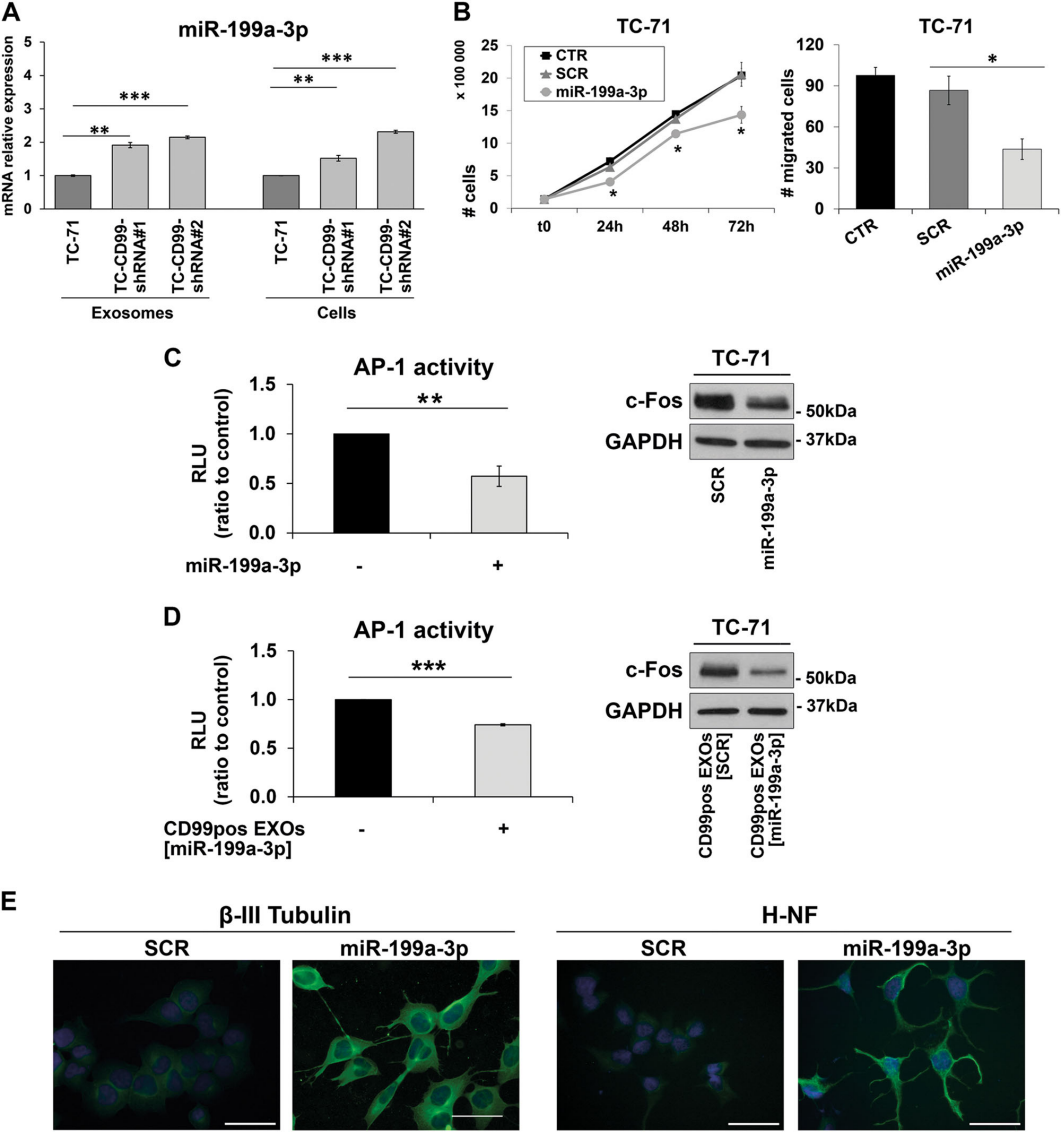
miR-199a-3p acts as an oncosuppressor in EWS, reducing tumor malignancy while inducing neural differentiation. **a** Validation of miR-199a-3p expression by qPCR in CD99neg- and CD99pos EXOs and their respective producing cells. The relative miRNA levels were normalized to the expression of miR-16 (for EXOs) or RNU6b (for cells). The data are shown as the mean ± SE of three independent experiments, with two replicates/each (**p < 0.01, ***p < 0.001, one-way ANOVA, *n* = 4). **b** miR-199a-3p mimic (30 nm) reduced cell growth (left; trypan blue vital count) and cell migration (right; transwell chambers) in TC-71 cells. The data are shown as the mean ± SE of three independent biological experiments with two duplicates/each (*p < 0.05, one-way ANOVA, *n* = 6). **c** miR-199a-3p mimic (30 nm) inhibited AP-1 transcriptional activity (as evaluated by luciferase assay) and c-Fos expression (as evaluated by western blotting) in TC-71 cells. The data are shown as the mean ± SE of three independent experiments with three replicates/each (**p < 0.01, Student’s *t*-test, *n* = 9). SCR, nonspecific control miRNAs. GAPDH was used as a loading control. **d** After transfection of miR-199a-3p mimic in TC-71, EXOs enriched for this miRNA were harvested (CD99pos EXOs [miR-199a-3p]) and used to treat parental EWS cells, TC-71. These enriched vesicles inhibited AP-1 transcriptional activity (as evaluated by luciferase assay) and c-Fos expression (as evaluated by western blotting) in TC-71 cells. The data are shown as the mean ± SE of at least three independent experiments (***p < 0.001, Student’s *t*-test). SCR, nonspecific control miRNAs. GAPDH was used as a loading control. **e** miR-199a-3p mimic (30 nm) induced neural differentiation. Representative immunofluorescence images from one experiment representative of two, showing β-III Tubulin expression (green) and H-NF expression (green) in TC-71 cells after exposure to miR-199a-3p. The nuclei were labeled with Hoechst 33258 (blue). Scale bars: 50 μm

The most enriched miRNA inside the CD99neg EXOs was miR-199a-3p. Through quantitative PCR (qPCR), we confirmed the increased expression of miR-199a-3p in CD99-deprived cell lines and in CD99neg EXOs (Fig. 6a, Supplementary Fig. 7a). Exposure of TC-71 and IOR/CAR cells to miR-199a-3p mimic inhibited cell growth and migration (Fig. 6b; Supplementary Fig. 7b, c), as was observed for CD99neg EXO exposure. Notably, in silico analysis with different bioinformatic algorithms (DIANA-MicroT and TargetScan) identified c-Fos as one of the predicted targets of miR-199a-3p; accordingly, we found inhibition of AP-1 activity and reduction in c-Fos levels (Fig. 6c, Supplementary Fig. 7d) together with induction of neural cell differentiation (Fig. 6e) in mimic-treated cells, replicating the observed effects of CD99neg EXOs (Fig. 4c). To reinforce our observations, we transfected the parental, CD99 positive TC-71 cells with the miR-199a-3p mimic in turn isolating EXOs enriched for the expression of this miRNA (named CD99pos EXOs [miR-199a-3p]). When TC-71 cells were exposed to these CD99pos EXOs [miR-199a-3p], effects on AP-1 activity and c-Fos expression (Fig. 6d) were found comparable to those obtained by direct miRNA mimic transfection (Fig. 6c). In addition, we analyzed the consequences on CD99-low EWS cells (TC-CD99-shRNA) derived from treatment with CD99pos EXOs enriched or not with miR-199a-3p (Supplementary Fig. 8). We focused on cell growth, migration and AP-1 activity and, in all the cases, the data indicated a specific action of miR-199a-3p with respect to control (scrambled = SCR).

To validate these observations in a clinical setting, we determined the expression levels of miR-199a-3p in a series of localized, untreated primary tumors (*n* = 62) in comparison with metastatic lesions (*n* = 51: 34 lung metastases and 17 bone metastases) from EWS patients. The expression of miR-199a-3p was significantly lower in metastases than in localized primary tumors (*p* < 0.05, Student’s *t*-test) (Supplementary Fig. 9), suggesting a relevant contribution of this miRNA to EWS aggressiveness.

## Discussion

EXOs, which are specialized extracellular nanovesicles ranging from 50 to 150 nm in diameter^17^, are now viewed as pivotal mediators in intercellular communication^18^ and have become the focus of exponentially growing interest as potential novel diagnostics or therapeutics. In cancer, EXOs may influence cancer cell behavior and the tumor microenvironment through instructions that are believed to be delivered by specific mRNAs, miRNAs and proteins that characterize EXOs' cargo^19–21^. Most investigations have explored the protumorigenic, proinvasive, and prometastatic effects of cancer cell-derived EXOs^22^. In this paper, we provide one of the few lines of evidence that cancer-derived EXOs may also have a detrimental effect on tumor cell aggressiveness. These oncosuppressor EXOs were obtained after silencing the cell surface molecule CD99 in the EWS cells. CD99 is a hallmark of EWS and is involved in several cellular processes, including cell adhesion, migration, death, and differentiation as well as intracellular protein trafficking, endocytosis, and exocytosis^23^. All EWS cells express the molecule at high levels, and CD99 is required for the maintenance of tumor aggressiveness. The molecule indeed prevents cell differentiation by communicating with EWS-FLI, the oncogenic driver of EWS, to maintain the proliferative and migratory as well as the metastatic capabilities of tumor cells^12,13^. Here, we confirmed that the reversion of malignancy and the induction of neural differentiation induced by genetic silencing of CD99 in EWS cells^12,13^ are replicated by the delivery of CD99neg EXOs to recipient EWS cells. EXOs derived from two EWS cell lines deprived of CD99 regulated gene expression in recipient cells and were able to inhibit cancer cell growth and migration while inducing cell differentiation in a panel of human patient-derived cell lines. These processes were oppositely regulated when CD99-deprived cells received EXOs derived from parental EWS cells, indicating that migration and differentiation in EWS cells are specifically regulated by CD99. Whether the presence or absence of this cell surface molecule determines the specific shuttling of particular cellular constituents into EXOs^24^ or whether EXOs packaging is just a reflection of the cell of origin^25,26^ is still unclear. Our data tend to favor the idea that EXOs reflect the experience of the cell and become relevant messengers and powerful propagators of cell status.

CD99neg EXOs were able to modulate the expression profile of EWS recipient cells, leading to up- or downregulation of genes relevant to EWS malignancy and neural differentiation. Among the top upmodulated genes (Supplementary Table 2) was the gene encoding galectin 3 binding protein (LGALS3BP), a matricellular protein with a role in tumor progression that has been shown to inhibit EWS proliferation, invasion, and metastasis^27^. Among the top downregulated genes were early growth response 1 (EGR1), a transcription factor that has been described to be downregulated in the nonmetastatic sarcoma cells^28^, and Jun/Fos, members of the AP-1 family of transcription factors^29^. AP-1 members are frequently overexpressed in sarcomas^30^, and c-Fos has been shown to specifically contribute to tumorigenesis and metastasis of bone sarcomas^31–33^. AP-1 lies downstream of many signaling pathways and transduces extracellular stimuli such as growth factors, cytokines, and environmental stresses, modulating a variety of biological processes including cell growth, death, differentiation, and oncogenic transformation^32^. The activity of the AP-1 complex can be regulated at many levels, including by constitutive protein expression changes, dimerization partners, phosphorylation, and protein–protein interactions with other transcription factors^34^. Here, we further investigated also the miRNA-specific content of CD99neg EXOs to identify the particular regulators that may be responsible for the modified profile of the recipient cells. The most differentially increased miRNA in CD99neg EXOs compared with CD99pos EXOs was miR-199a-3p; quite interestingly, c-Fos is among the predicted targets of this miRNA. Exposure of EWS cells to miR-199a-3p mimic as well as to miR-199a-3p-enriched EXOs indeed replicated the phenotypic effects of CD99neg EXOs, including inhibition of AP-1 activity and the expression of its target genes (i.e., MMP9, MMP1, and CCND1). miR-199a-3p has been reported to suppress tumor growth, migration, invasion, and metastasis while increasing chemosensitivity in several tumors, including osteosarcoma^35,36^; in addition, it has been described as an independent marker of improved survival in bladder cancer^37^. In this paper, we confirmed that miR-199a-3p is decreased in metachronous metastases compared with primary tumors, supporting its clinical relevance even in EWS. This indicates that the effects of CD99neg EXOs may be mediated, at least in part, by enhanced delivery of this miRNA. Nevertheless, other than miR-199a-3p miRNAs might be relevant as well. Indeed, we identified a signature composed of 56 differentially expressed miRNAs whose impact on EWS cell behavior needs further consideration.

**Fig. 7:**
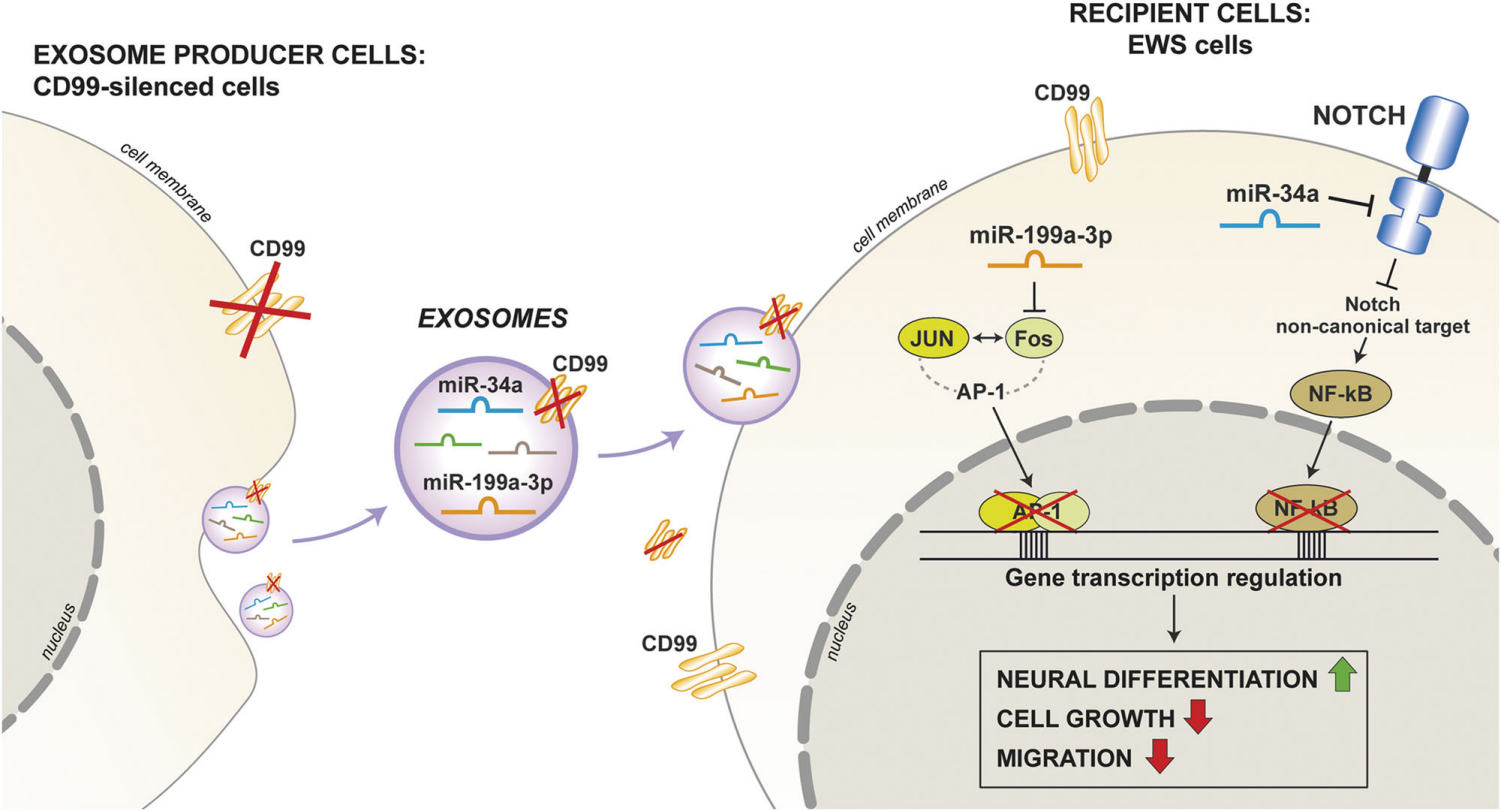
Schematic representation of the main molecular mechanisms that link CD99-deprived EXOs with malignant reversion of EWS cells. The figure depicts the role of two oncosuppressor microRNAs, which were enriched in CD99neg EXO cargo: miR-199a-3p and miR-34a, the first acting through AP-1 activity, the former exerting its action by means NOTCH/NF-kB axis. They both contribute to the reversion of malignancy resulting in the induction of neural differentiation together with the decrease of cell growth and migration

In addition, our previous observations have highlighted an important role also for the miR-34a^13^. Considering that miRNAs have multiple targets and their action is strictly interconnected, it is conceivable that even minimal fluctuations in the expression of multiple miRNAs will result in substantial changes in the mechanisms of tumor progression. A schematic representation of the so far described molecular connections that link CD99-deprived EXOs with malignant reversion of EWS cells is shown in Fig. 7.

Taken together, we have shown that modulating the expression of a fundamental cell surface molecule in EWS cells leads to the release of EXOs delivering a specific group of miRNAs with oncosuppressor functions that may be transferred to other tumor cells and substantially reduce malignancy. Development of best-practice models for the therapeutic use of EXOs is still a challenge^38^, but the use of EXOs as “natural” nanocarriers of anticancer therapies has gained growing interest, and engineering of EXOs is an active field offering new therapeutic opportunities to control primary tumors and metastatic disease^39^. Here, we provide a novel conceptual therapeutic approach against a tumor type that remains refractory to most treatments except conventional chemotherapy.

## Materials and methods

### Cell lines and primary cultures

EWS cell lines were grown as previously described^13,40^. The lines 6647 and TC-71 were kindly provided by T.J. Triche (Children's Hospital, Los Angeles, CA, USA); SK-N-MC, SK-ES-1, and RD-ES were provided by American Type Culture Collection, ATCC (Rockville, MD, USA); IOR/CAR and LAP-35 were previously established in our laboratory; and the A673 sarcoma cell line was provided by Dr. H. Kovar (St. Anna Kinderkrebsforschung, Vienna Austria). Stable CD99-silenced cells were obtained from the TC-71 and IOR/CAR cell lines as previously described^12,13^. The cells were cultured in Iscove's modified Dulbecco's medium (IMDM; EuroClone, Milan, Italy) enriched with 10% fetal bovine serum (FBS) (EuroClone) supplemented with 100 U/ml penicillin and 100 mg/ml streptomycin and incubated at 37 °C in a humidified atmosphere at 5% CO_2_.

All cell lines were tested for mycoplasma contamination (MycoAlert Mycoplasma Detection Kit, Lonza) and authenticated by short tandem repeat (STR) polymerase chain reaction (PCR) analysis (last control: December 2017) using a PowerPlex ESX Fast System kit (Promega, Madison, WI, USA).

### Isolation, characterization and labeling of EXOs

EXOs were isolated from cell culture medium with the EQ (System Biosciences, Mountain View, CA, USA) or by ultracentrifugation (UC) methods according to the manufacturer’s instructions or standard procedures^15^.

In EQ, briefly, cells were cultured for 24 h in IMDM supplemented with EXO-depleted FBS. The serum was depleted of bovine EXOs by UC at 100,000 × *g* for 6 h, followed by filtration through a 0.2 µm filter prior to use. The conditioned medium was centrifuged at 3000 × *g* for 15 min to remove cell debris. The supernatant was transferred to a new sterile tube and EQ precipitation solution was added for an overnight incubation at +4 °C. EXOs were isolated the day after by centrifugation at 1500 × g for 30 min at room temperature (RT). The EXO pellets were then resuspended in IMDM 0% FBS for in vitro functional studies, in Trizol for RNA extraction or in lysis buffer for western blot. The protein concentration of the EXOs was determined using a protein assay kit (Bio-Rad, Hercules, CA, USA), and in some cases, the number and size of the EXOs were directly tracked using a NanoSight NS300 system (NanoSight technology, Malvern, UK), configured with a 488 nm laser and a high-sensitivity sCMOS camera. Videos were collected and analyzed using NanoSight NTA software (version 3.0). For each sample, multiple videos 60 s in duration were recorded to generate replicate histograms, which were averaged.

The fatty acid molecule BODIPY® FL C16 (16 μm) was included in the culture medium to label EWS cells, as it is incorporated into the cell membranes. EXOs (10 µg) were cocultured with 5,000 recipient cells grown in eight-well chamber slides (Falcon, NY, USA). After 3 h of incubation, the cells were fixed in 4% paraformaldehyde (Sigma-Aldrich, St Louis, MO, USA) for 10 min. Cell staining was analyzed with a Nikon A1R confocal microscope with a Plan Apo 60 ×/NA 1.4 DIC N2 objective. The uptake of fluorescent vesicles in recipient cells was assessed by flow cytometric analysis as previously reported^41^.

### In vitro functional assays

EXOs in equal amounts (10 µg/5,000 cells), purified from either parental or CD99-silenced EWS cell lines, were incubated in serum-free conditions with recipient cells at 37 °C before functional studies were performed.

### Cell viability assessment with trypan blue dye

A total of 50,000 cells/well were seeded in 24-well plates. CD99neg- or CD99pos EXOs were added after 24 h. The cells were exposed to the EXOs for up to 48 h before being harvested and counted with trypan blue vital cell dye (Sigma-Aldrich). Cells that were not treated were used as controls.

### Cell proliferation assessment with Ki-67 staining

The EWS cell lines were exposed to CD99neg EXOs 48 h and then fixed with methanol for 7 min at − 20 °C. The slides were incubated with a primary antibody against Ki-67 (Mib1; Sigma-Aldrich) overnight at +4 °C and then with an anti-mouse fluorescein isothiocyanate (FITC) secondary antibody (Thermo Fisher Scientific, Waltham, MA, USA) for 30 min at RT. The nuclei were counterstained with Hoechst 33258 (0.5 μg/ml; Sigma-Aldrich).

### Cell death analysis

EWS cell lines (TC-71, IOR/CAR, and LAP-35) were treated with CD99neg EXOs for 48 h. Detection and quantification of apoptotic cells were performed by flow cytometric analysis (FACSCalibur; Becton Dickinson) of Annexin-V-FITC-labeled cells, according to the manufacturer's instructions (MEBCYTO Apoptosis Kit, Annexin-V FITC Kit, MBL Life Science). Propidium iodide (PI) incorporation was evaluated in association with the fluorescent signal intensity to allow the discrimination between necrotic and apoptotic cells.

### Migration assay (transwell chambers)

Migration ability in the EWS cell lines was assessed using transwell chambers (Costar, Cambridge, MA) with an 8 μm pore size and polycarbonate filters. A cell suspension (100,000 cells) was pretreated with CD99neg- or CD99pos EXOs for 30 min in serum-free IMDM at 37 °C. The cells were seeded in the upper compartment, whereas IMDM plus 10% FBS was placed in the lower compartment of the chamber as a chemoattractant. Incubation was performed overnight at 37 °C in a humidified atmosphere. The migrated cells were fixed with methanol, stained with Giemsa dye and counted at 10 × magnification. A673 cells had a low capability to migrate, so they were seeded in 1% FBS in the upper compartment, and IMDM 20% FBS was placed in the lower compartment (gradient).

### Wound-healing assay

EWS cells were seeded in 24-well plates coated with fibronectin (3 μg/cm^2^; Sigma-Aldrich). The cells were allowed to grow until 100% confluence was achieved. A pipette tip was used to obtain the cell free lane and the medium was then renewed and supplemented with purified EXOs from TC-CD99-shRNA or CAR-CD99-shRNA cells or the parental cell line TC-71. Images were obtained at time 0 and after 24 h under an inverted microscope (Zeiss, Inc., Thornwood, NY).

### Neural differentiation

To assess neural differentiation in EWS cultures, cells were plated at low densities (5,000 cells/cm^2^) in standard medium. The cells were then exposed to purified CD99neg EXOs for 48 h. Immunofluorescence was performed on adherent cells grown on coverslips for 72 h, fixed in methanol/acetone 3:7 or 4% paraformaldehyde, permeabilized with 0.15% Triton X-100 in phosphate-buffered saline (PBS) and incubated with anti-heavy neurofilament (H-NF, cat. #2836; Cell Signaling Technology, Beverly, MA, USA) and anti-β-III Tubulin (cat. #T5076; Sigma-Aldrich) antibodies. The nuclei were counterstained with bisbenzimide (Hoechst 33258; Sigma-Aldrich). Cell fluorescence was then evaluated using a Nikon Eclipse 90i microscope (Nikon Instruments, Florence, Italy) equipped with a Plan Apo VC 60 oil, NA 1.4 objective at RT. Images were captured under identical conditions using a digital camera (Nikon DS5MC) and the software NIS‐Elements AR 3.10 (Nikon). The same software was used to merge all images with double labeling. For the neurite outgrowth assay, cells were classified as differentiated if they exhibited an outgrowth extending from the cell that was at least 1.5 times the diameter of the cell. At least 100 cells were counted on each slide.

### Luciferase reporter gene assay

A luciferase assay was used to evaluate the transcriptional activity of AP-1. Cells were seeded in standard medium in 24‐well plates (30,000 cells/well) previously coated with fibronectin (3 μg/cm^2^; Sigma-Aldrich). Transfection was performed 24 h later with 250 ng of AP-1 signal reporter (Qiagen, Hilden, Germany) using Lipofectamine 2000 (Thermo Fisher Scientific) according to the manufacturer’s instructions. At 24 h after transfection, the same amounts of CD99neg EXOs were incubated with recipient cells for 30 min at 37 °C before assessment of luciferase activity. The cells were lysed, and luciferase activity was measured according to the manufacturer’s protocol using a Dual‐Glo Luciferase Assay System (Promega) with a GloMax Luminometer (Promega).

The firefly luciferase luminescence signal was normalized to that produced by Renilla luciferase, which was included as an internal control. The data are represented as relative light units (RLU).

### Immunoblotting analysis

Western blot experiments were performed according to standard protocols. Samples were lysed in radioimmunoprecipitation assay (RIPA) buffer (50 mm Tris-HCl, pH 7.4, 150 mm NaCl, 0.1% sodium dodecyl sulfate (SDS), 1% Triton X-100, 5 mm ethylenediaminetetraacetic acid (EDTA), 1% deoxycholate) supplemented with protease inhibitors.

Equivalent amounts of lysates were run on SDS gels under denaturing conditions and blotted onto nitrocellulose membranes. The membranes were incubated overnight with anti-CD99 (12E7; sc-53148; Santa Cruz Biotechnology, Santa Cruz, Dallas, USA), anti-GAPDH (sc-25778; Santa Cruz Biotechnology), anti-c-Fos (9F96; #2250; Cell Signaling Technology) primary antibodies. Anti-rabbit or anti-mouse antibodies conjugated to horseradish peroxidase (GE Healthcare, Little Chalfont, UK) were used as secondary antibodies. The proteins were visualized with an ECL Western Blotting Detection System (GE Healthcare). The signal for CD99 protein was quantified by using GS-800 imaging densitometer (Bio-Rad, Hercules, CA) and the Quantity One 4.6.9 software (Bio-Rad). The values are represented as optical Density (OD).

### RNA extraction and qPCR

RNA from EXOs was isolated using miRNeasy columns (Qiagen) according to the manufacturer’s protocol; total RNA from cell lines was extracted with a TRIzol extraction kit (Life Technologies, Grand Island, NY, USA). Nucleic acid quality and quantity were assessed with a NanoDrop spectrophotometer (NanoDrop Technologies, ThermoFisher Scientific). Total RNA for each sample was reverse transcribed into cDNA using a High-Capacity cDNA Reverse Transcription Kit (Life Technologies) according to the manufacturer’s protocols. qPCR was performed on a ViiA7 system (Life Technologies) using TaqMan PCR Master Mix (Life Technologies). Predesigned TaqMan probes (Life Technologies) were used for Matrix Metalloproteinase 1 (MMP1, Hs00899658_m1), Matrix Metalloproteinase 9 (MMP9, Hs00957562_m1), Cyclin D1 (CCND1, Hs00765533_m1), CD99 (Hs00908458) and miR-199a-3p (Hs002304). Relative quantification as performed with the ΔΔCT method, and the expression levels of the target genes were normalized to those of the housekeeping gene GAPDH (Hs99999905_m1), RNU6b (Hs001093) or miR-16 (Hs000391).

### Microarray profiling and data analysis

Gene expression patterns were assessed using a GeneChip® Human Transcriptome Array 2.0 (Affymetrix, Santa Clara, CA, USA) starting from 100 ng of total RNA. Microarray data are available at the GEO database (https://www.ncbi.nlm.nih.gov/geo) with the accession number # GSE128681. The microarray target sample processing, target hybridization, washing, staining, and scanning steps were completed according to the manufacturer's instructions (Affymetrix). Raw data were analyzed with GeneSpring software v.14.9 (Agilent Technologies, Santa Clara, CA, USA). Specifically, .CEL files were imported and processed using the ExonRMA16 summarization algorithm. Quantile normalization was applied without baseline transformation. Differentially expressed genes were defined as having a ≥ 1.5-fold expression difference between groups and an adjusted *p*-value of less than 0.05 in a moderated *t-*test with Benjamini and Hoechberg correction for false positive reduction. Hierarchical clustering was performed for the TC-71 samples with the GeneSpring clustering tool using Pearson’s centered correlation as a measure of similarity. Pathway and network analyses of the differentially expressed genes were performed using the web-based software MetaCore (GeneGo, Thomson Reuters).

### microRNA profiling and data analysis

miRNA expression was investigated using an Agilent Human miRNA Microarray #G4870C and Sanger miRBase v.21 (Agilent Technologies). Microarray data are available at the ArrayExpress database (https://www.ebi.ac.uk/arrayexpress) with the accession number E-MTAB-7585. RNA labeling and hybridization were performed in accordance with the manufacturer’s instructions. The microarray results were analyzed with GeneSpring software v.14.9 (Agilent Technologies). Quantile normalization and log2 transformation were applied. Differentially expressed miRNAs were selected that had a 1.5-fold difference between CD99pos- and CD99neg EXOs and an adjusted *p* value < 0.20 in a moderated *t*-test with Benjamini and Hoechberg correction. Hierarchical clustering was performed using Pearson’s centered correlation as a measure of similarity.

### Flow cytometry

CD99 expression was evaluated by flow cytometry in EXOs isolated with EQ or by UC^15^. We used a staining protocol involving latex beads (aldehyde/sulfate latex #A37306; Invitrogen) that were incubated with 20 μg of EXOs for 30 min at RT. Bovine serum albumin (BSA, 1%) was added to the samples, and the mixture was incubated overnight at 4 °C on a test tube rotator. After three washes in PBS with 0.5% BSA, the EXOs-bead mixture was incubated with an anti-CD99 antibody (12E7; sc-53148; Santa Cruz) for 30 min at 4 °C and with an anti-mouse FITC secondary antibody (#31569; Thermo Scientific). The data were acquired using a BD FACS LSR III (BD Transduction Laboratories, Lexington, KY, USA).

CD99 levels were analyzed in EWS cells with a FACSCalibur flow cytometer (BD Transduction Laboratories). The antibodies used were anti-CD99 O13 (Signet Laboratories, Dedham, MA, USA) and an anti-mouse FITC secondary antibody (Thermo Scientific).

### miR-199a-3p transfection

Twenty-four hours after cell seeding, cells were transfected with pre-miR-199a-3p mimic or with nonspecific control miRNAs (SCR) (30 nm) (assays #AM17100 and #AM17110; Thermo Scientific) using a *Trans*IT-X2 Dynamic Delivery System (Mirus, Madison, WI, USA). The expression level of miR-199a-3p was determined by qPCR up to 48 h after transfection. Functional studies were performed as previously described for the CD99neg- and CD99pos EXO treatments.

In parallel, from the transfected TC-71 cells, EXOs enriched for miR-199a-3p were harvested and used for biological assays in TC-71 cells and CD99-deprived cells, TC-CD99-shRNA. The experiments performed reproduce the setting previously described in this section of material and methods.

### Clinical samples

A cohort of 113 patients with confirmed diagnosis of EWS treated at the Istituto Ortopedico Rizzoli (Bologna, Italy) was considered. The EWS patients underwent homogenous treatments based on local control of the disease (surgery and/or radiotherapy, depending on the tumor site) and systemic neoadjuvant chemotherapy. All the patients were enrolled in clinical studies, previously reported in detail approved^42,43^. miR-199a-3p expression was evaluated by qPCR in localized primary tumors (62 cases) or metachronous metastases that developed from 6 months to 10 years after the end of treatments (51 cases: 17 bone and 34 lung). The ethical committee of the Istituto Rizzoli approved the study (0019012/2016), and informed consent was obtained. The study was conducted in accordance with the Declaration of Helsinki ethical guidelines.

### Statistical analyses

All statistical analyses were performed using Prism version 7.0 (GraphPad Software, La Jolla, CA). Differences among means were evaluated by one-way analysis of variance (ANOVA) with Tukey’s multiple comparisons test, whereas two-tailed Student’s *t-*test was used for comparisons between two groups. The data were considered statistically significant at *p* < 0.05. Pearson’s test was used to evaluate correlations.

## Supplementary information

Supplementary Figures

Supplementary Table 1

Supplementary Table 2

Supplementary Table 3

Supplementary Table 4

original blots

Supplementary figure legends
