# Supplementary material for: Exosomes from CD99-deprived Ewing sarcoma cells reverse tumor malignancy by inhibiting cell migration and promoting neural differentiation
Source: Cell Death Dis. 2019 Jun 17;10(7):471. doi: 10.1038/s41419-019-1675-1 (PMC6572819; doi:10.1038/s41419-019-1675-1)
Supplement: Supplementary file 1 — Supplementary Figures [file 41419_2019_1675_MOESM1_ESM.pdf]

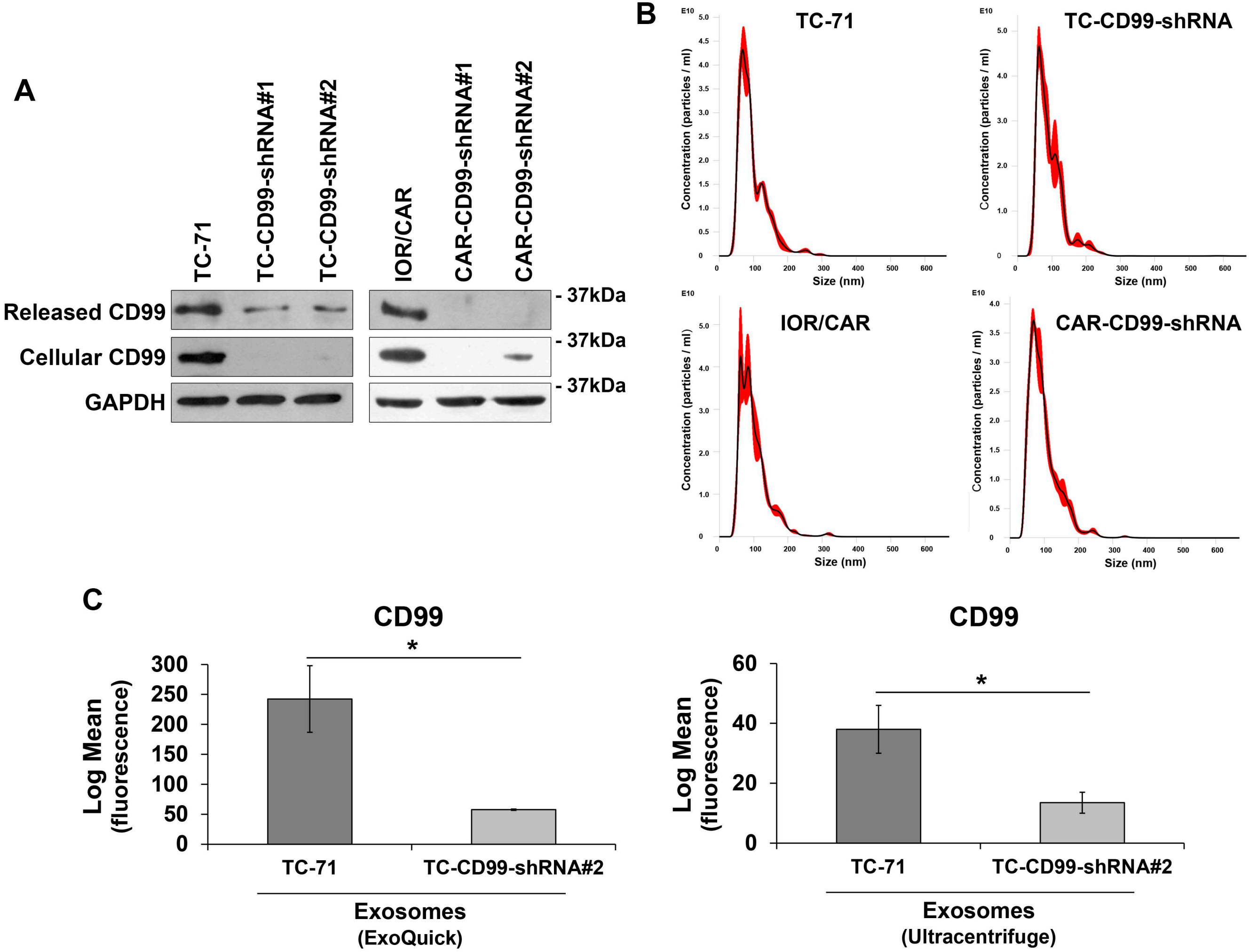

Supplementary Figure 1

a Representative western blot showing CD99 secreted in the supernatants of TC-CD99-shRNA and CAR-CD99-shRNA experimental models. CD99 protein expression from productive cells is shown in comparison. GAPDH was used as a loading control. **b** Size distribution of vesicles derived from EWS cells lines and CD99-silenced cells (TC-71 and TC-CD99-shRNA, IOR/CAR and CAR-CD99-shRNA) analyzed by the Nanosight ™ technology. **c** CD99 expression evaluated by flow cytometry on the surface of EXOs from TC-71 and TC-CD99-shRNA cells. The two graphics show in comparison results from vesicles isolated by ExoQuick® or by Ultracentrifuge (*p<0.05, unpaired Student's t-test).

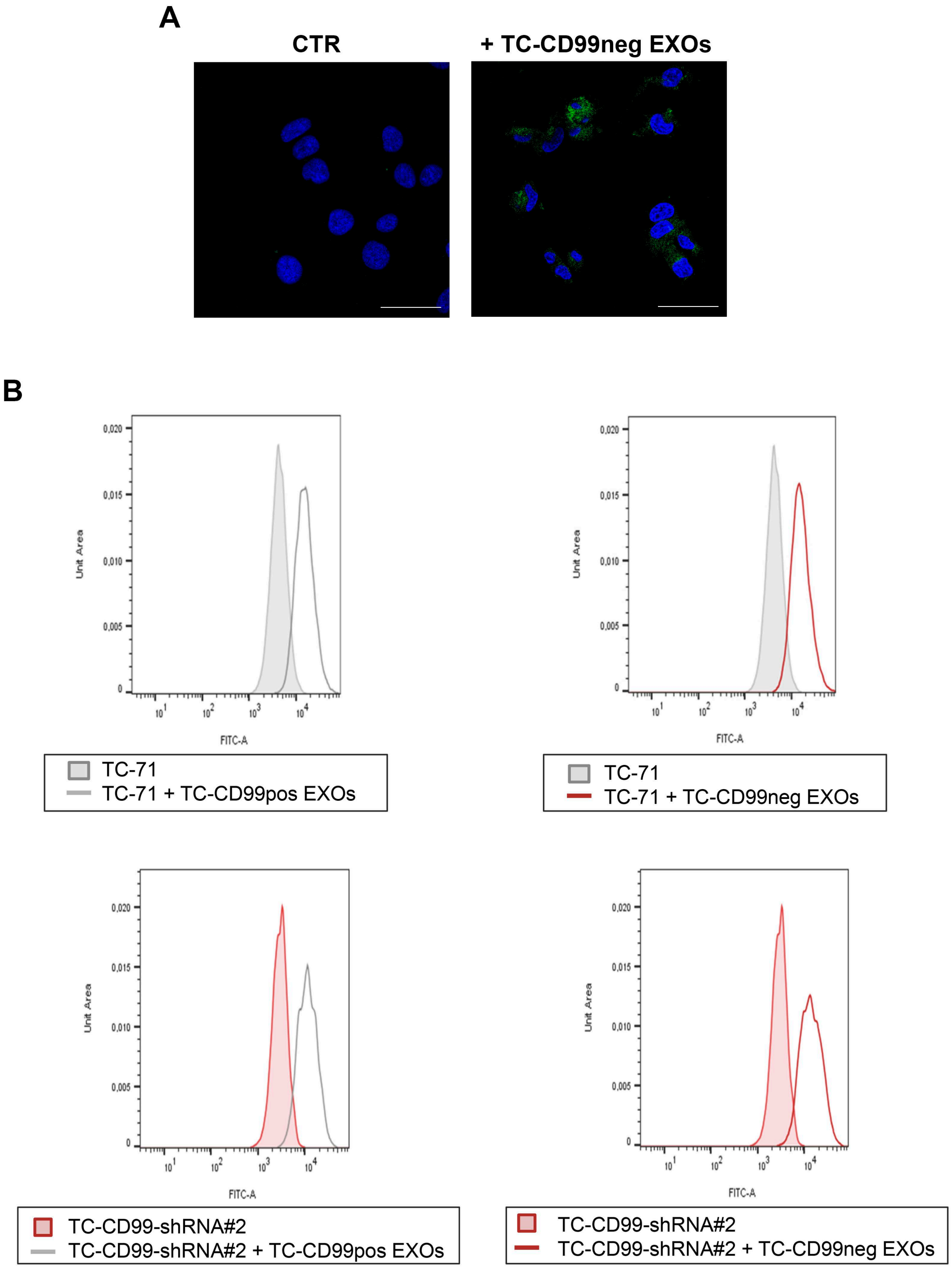

Supplementary Figure 2

a Confocal microscopy image of exosome uptake in untreated TC-71 cells and TC-71 cells treated with CD99neg EXOs. Internalized vesicles, which were labeled with BODIPY FL C16, were evidenced by a green fluorescent punctuate signal inside cytoplasm of TC-71 recipient cells. The nuclei were counterstained with Hoechst. Scale bar: 50 μm.

b FACS analysis on TC-71 and CD99-silenced cells after exosome exposure. The fluorescent signal confirmed the similar uptake of CD99neg and CD99pos vesicles in recipient cells.

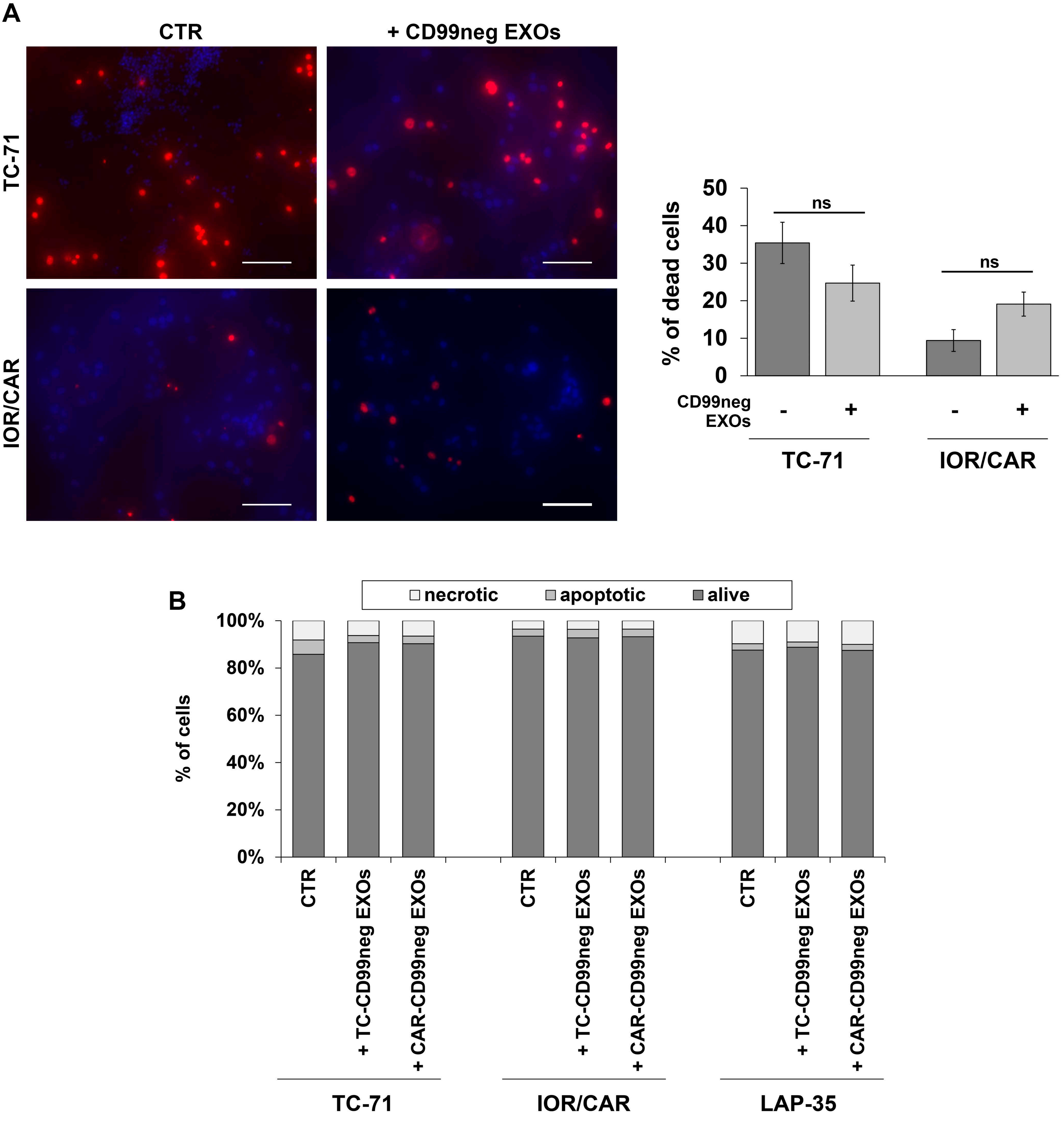

Supplementary Figure 3

a Immunofluorescence analysis of PI staining in TC-71 and IOR/CAR cell lines receiving or not receiving (CTR) CD99neg EXOs. The cells were counterstained with vital Hoechst 33258 (blue). Scale bar: 100 μm. PI-positive cells were counted, and percentages of positive cells are shown on the right panel as the mean±SE (ns=not significant, Student's *t*-test). **b** Evaluation of apoptosis by Annexin-V staining in EWS cells treated or not treated (CTR) with CD99neg EXOs.

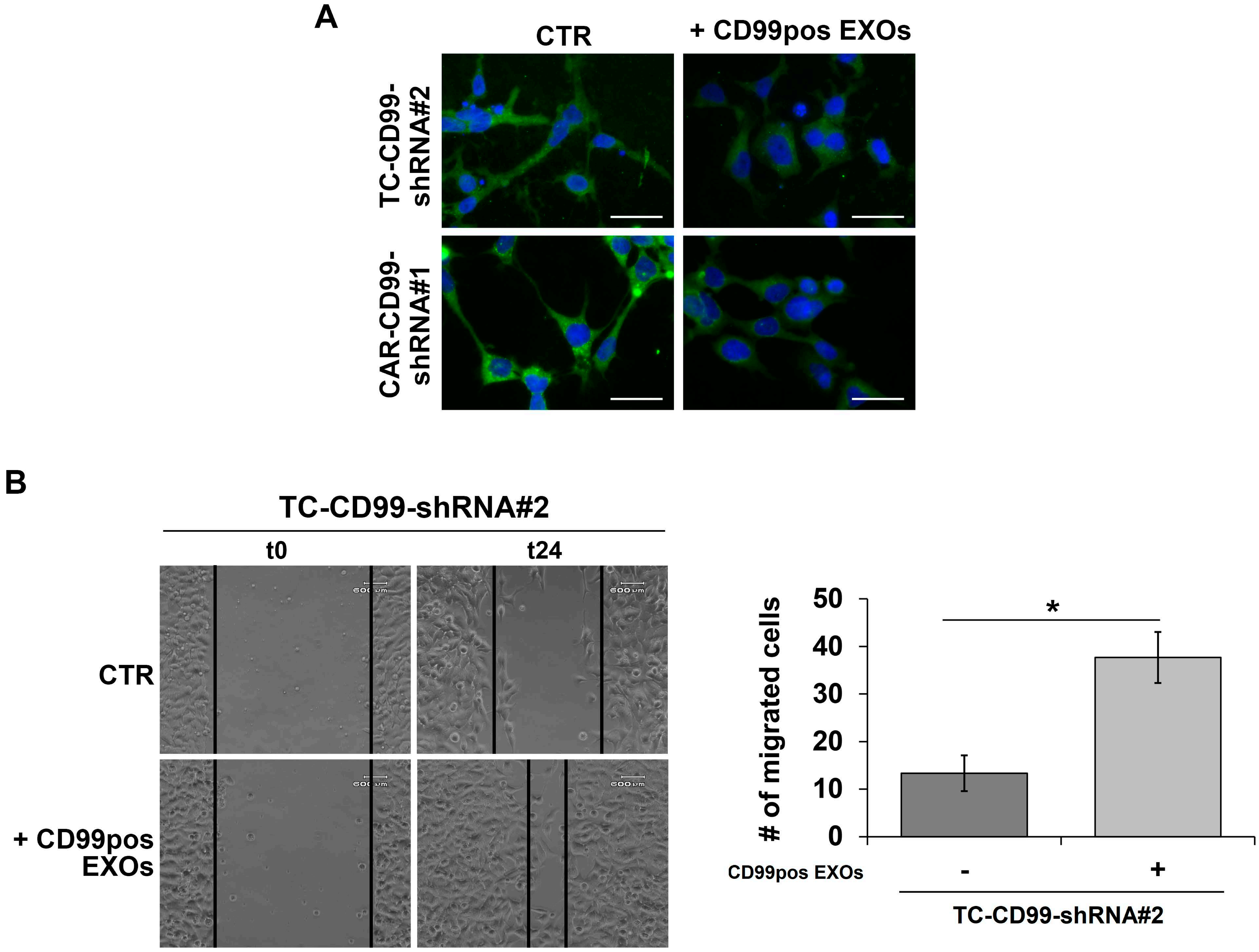

Supplementary Figure 4

a Immunofluorescence staining of β-III Tubulin (green) in stable CD99-silenced clones (TC-CD99-shRNA#2 and CAR-CD99-shRNA#1), receiving or not receiving (CTR) CD99pos EXOs derived from their respective parental cell lines. The nuclei were labeled with Hoechst 33258 (blue). Representative images are displayed. Scale bars: 50 μm. **b** Cell migration of TC-CD99-shRNA#2 cells exposed or not exposed (CTR) to CD99pos EXOs. Results of wound-healing assays (left) and motility assays with Transwell chambers (right). The data are shown as the mean±SE (*p<0.05, Student's *t*-test).

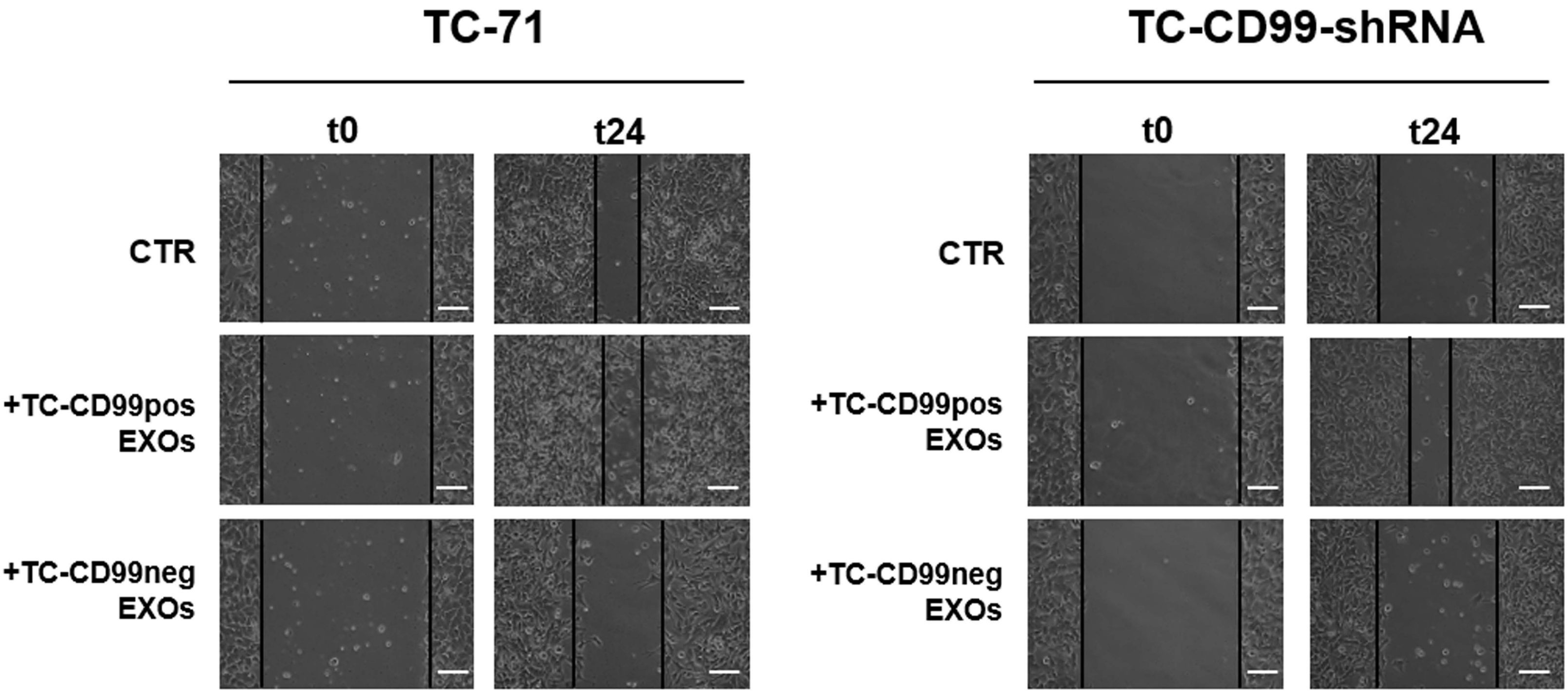

Supplementary Figure 5

Wound healing was performed in EWS cells, TC-71 and the CD99-silenced variant TC-CD99-shRNA. Each cell line was treated or not (CTR) with both CD99pos- or CD99 neg EXOs. Scale bars: 100 μm.

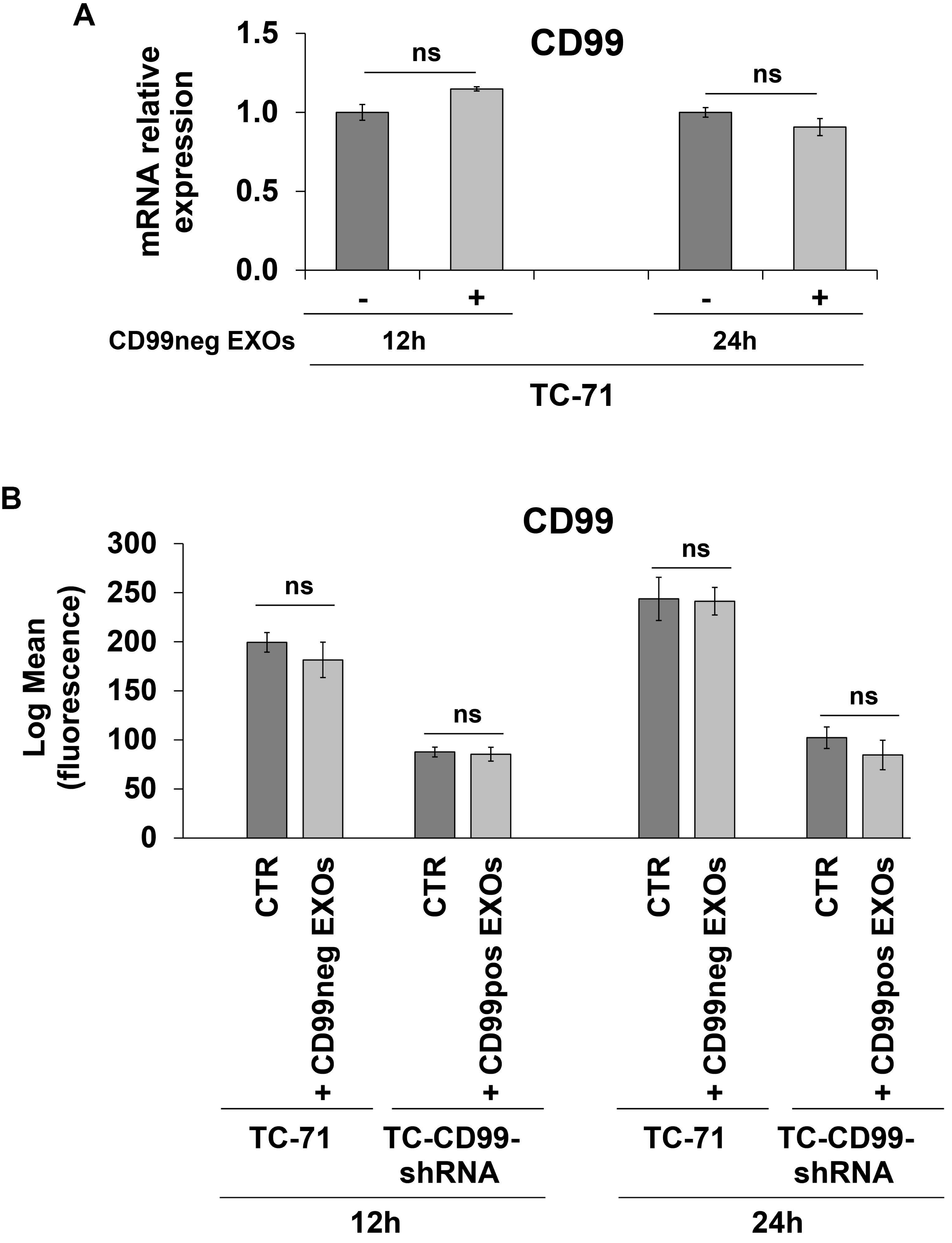

Supplementary Figure 6

a qPCR results showing the relative expression of CD99 in TC-71 cells receiving or not receiving (CTR) CD99neg EXOs for 12 h or 24 h. The mRNA levels were quantified with the $2^{-\Delta\Delta Ct}$ method. Untreated cells were used as calibrators ($2^{-\Delta\Delta Ct}$=1), and GAPDH was used as a housekeeping gene (ns= not significant, Student's *t*-test). **b** Evaluation of CD99 expression by flow cytometry in TC-71 or TC-CD99-shRNA#2 cells treated or not treated (CTR) with CD99neg or CD99pos EXOs for 12 h or 24 h (ns= not significant, Student's *t*-test).

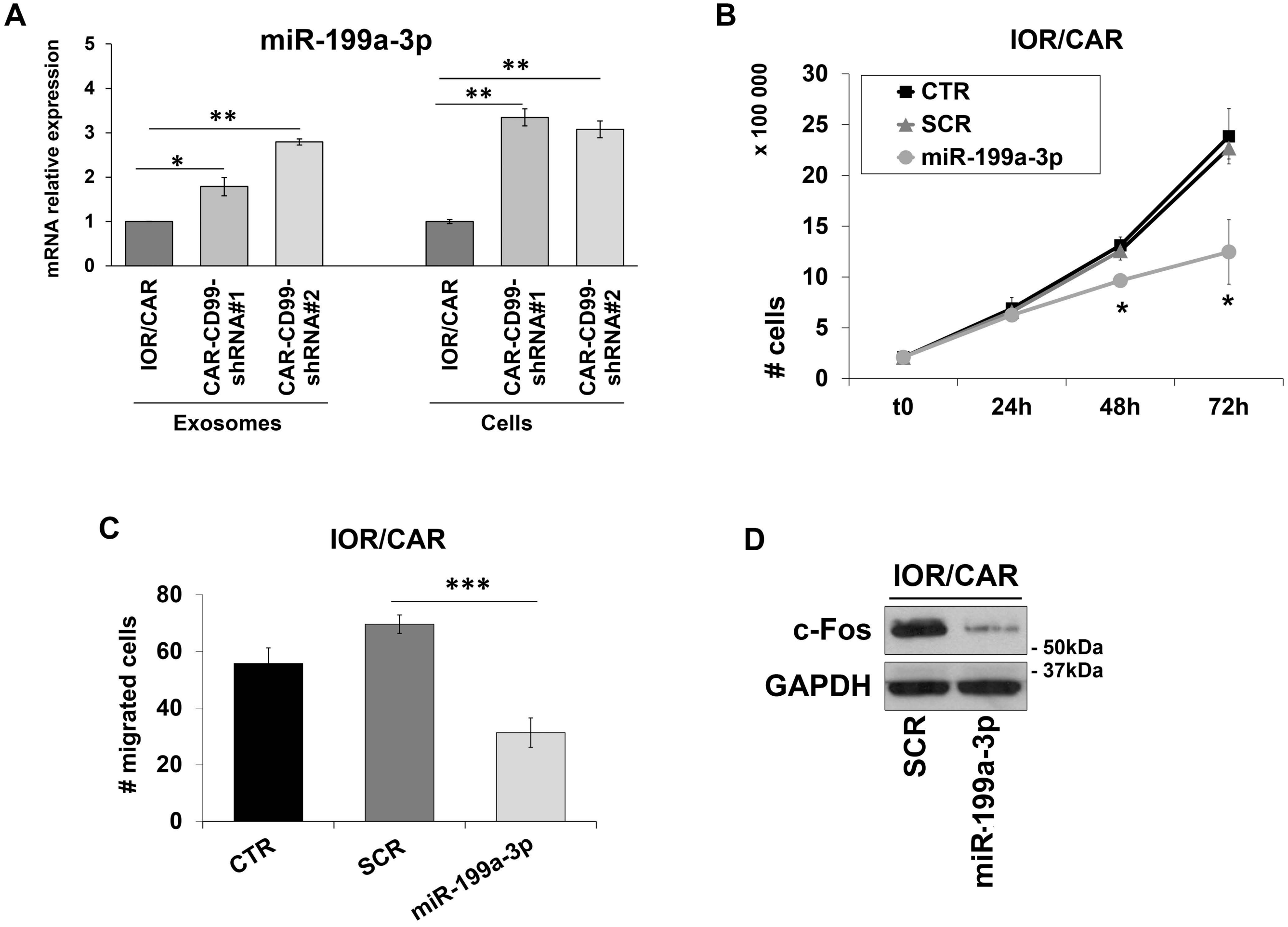

Supplementary Figure 7

a Validation of miR-199a-3p expression by qPCR in CD99neg and CD99pos EXOs and their respective producing cells. The relative miRNA levels were normalized to the expression of miR-16 (for EXOs) or RNU6b (for cells). The data are shown as the mean±SE (*p<0.05, **p<0.01, one-way ANOVA). miR-199a-3p mimic (30 nM) reduced cell growth (trypan blue vital count, **b**) and cell migration (transwell chambers, **c**) in IOR/CAR cells. The data are shown as the mean±SE of at least three independent experiments (*p<0.05, ***p<0.001, one-way ANOVA). **d** miR199a-3p mimic (30 nM) decreased c-Fos expression (as evaluated by western blotting) in IOR/CAR cells. SCR, nonspecific control miRNAs. GAPDH was used as a loading control.

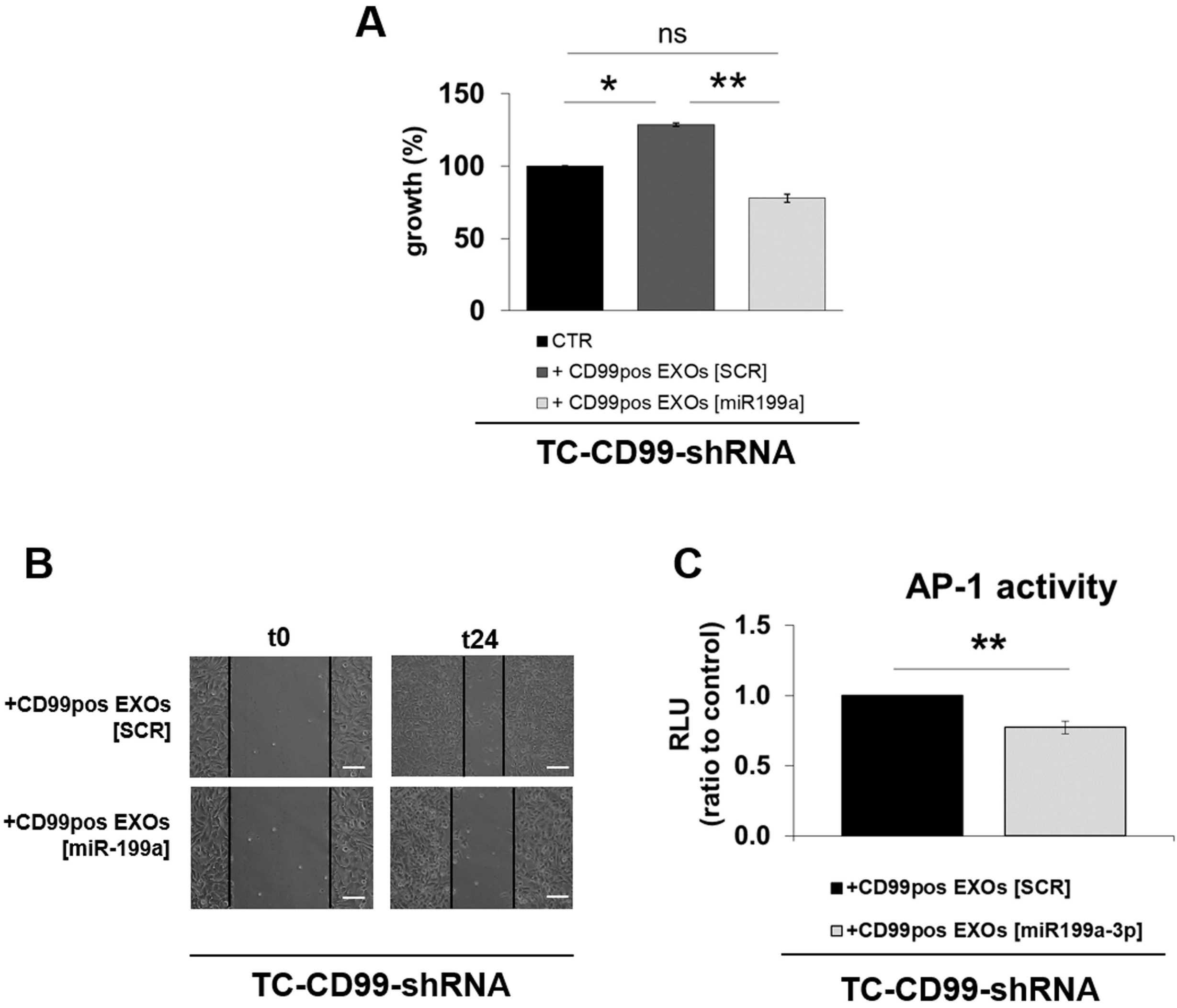

Supplementary Figure 8

After transfection of miR-199a-3p mimic in TC-71 cells, exosomes enriched with this miRNA were harvested (CD99pos EXOs [miR-199a-3p]) and used to treat CD99-deprived cells, TC-CD99-shRNA. The following parameters were analyzed:

a Cell growth by trypan blue count. Columns show the mean values ± SE (*p<0.05, **p<0.01, One-way ANOVA).

b Migration by wound healing assay. Scale bars: 100 μm.

c AP-1 transcriptional activity by luciferase assay. Columns represent the mean values ± SE (**p<0.01, Student's t-test). Scrambled (SCR) mimic was used as a transfection control.

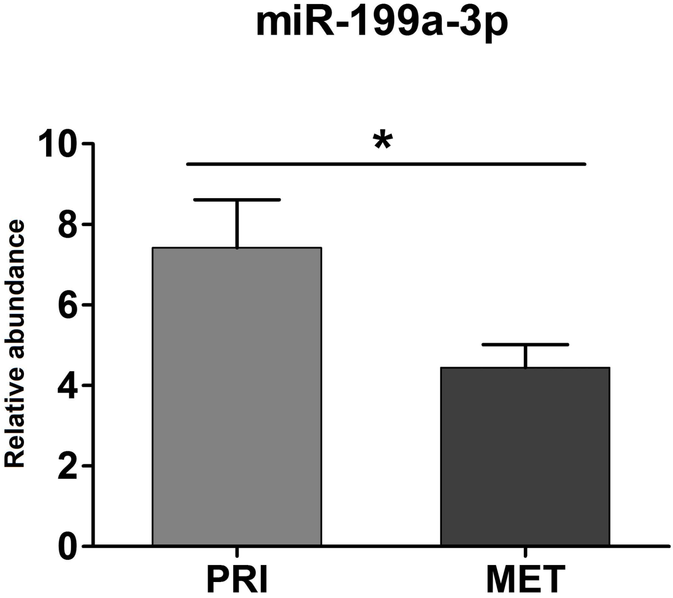

Supplementary Figure 9

Relative expression of miR-199a-3p in EWS clinical samples as determined by qPCR (mean ± SE).

Primary tumors (n=62) and metastases (n=51) were compared. The data were quantified with the $2^{-\Delta CT}$ method. RNU6b was used as a housekeeping gene (*$p<0.05$, Student's *t*-test).
