# Supplementary material for: Exosomes from CD99-deprived Ewing sarcoma cells reverse tumor malignancy by inhibiting cell migration and promoting neural differentiation
Source: Cell Death Dis. 2019 Jun 17;10(7):471. doi: 10.1038/s41419-019-1675-1 (PMC6572819; doi:10.1038/s41419-019-1675-1)
Supplement: Supplementary file 6 — original blots [file 41419_2019_1675_MOESM6_ESM.pdf]

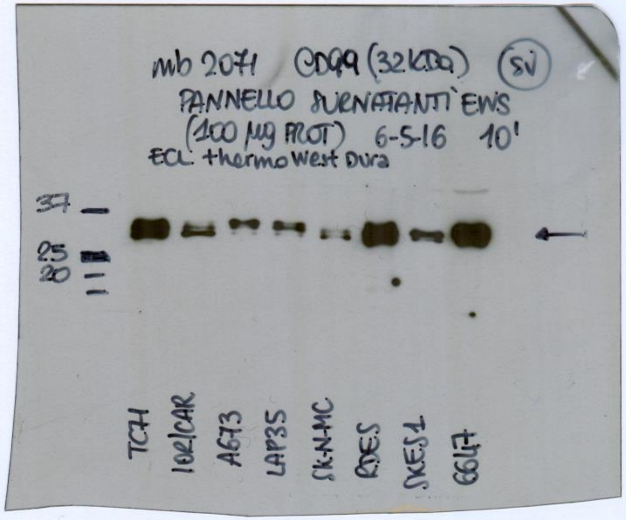

Full unedited gel for **Figure 1**

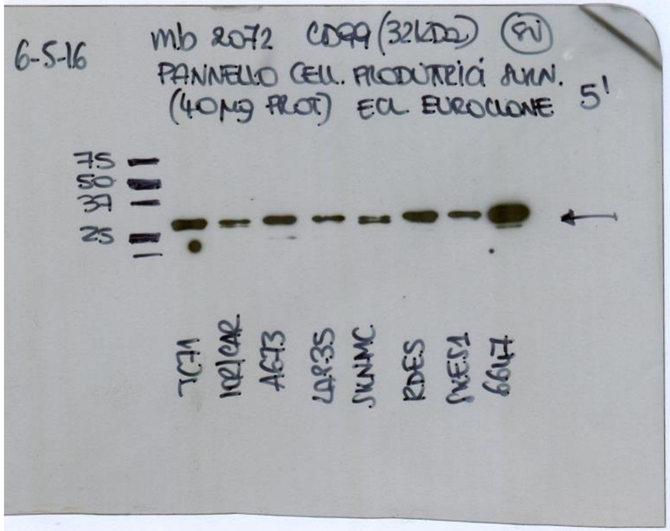

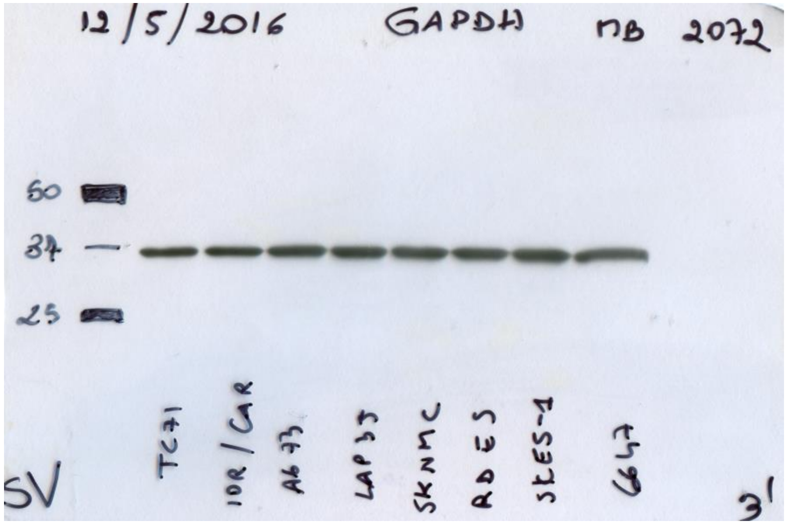

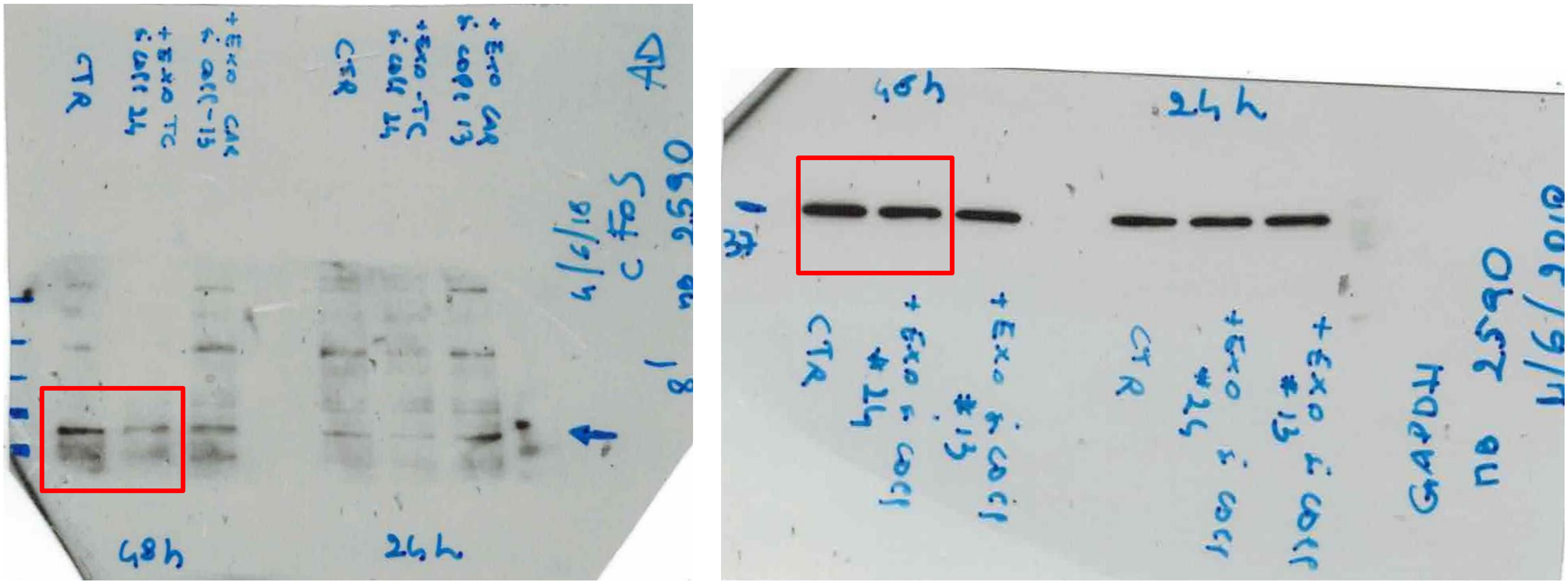

Full unedited gel for **Figure 4**

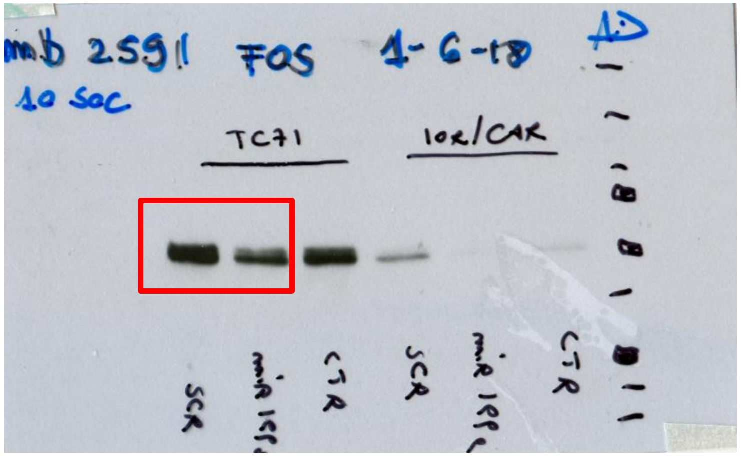

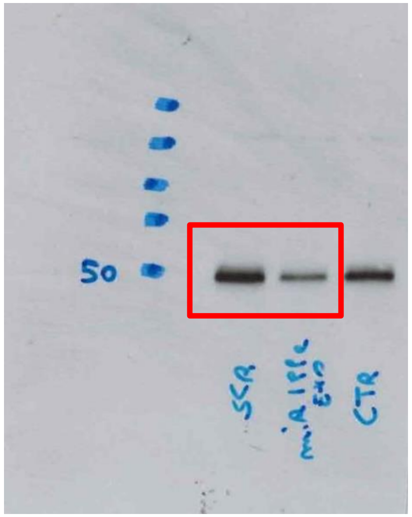

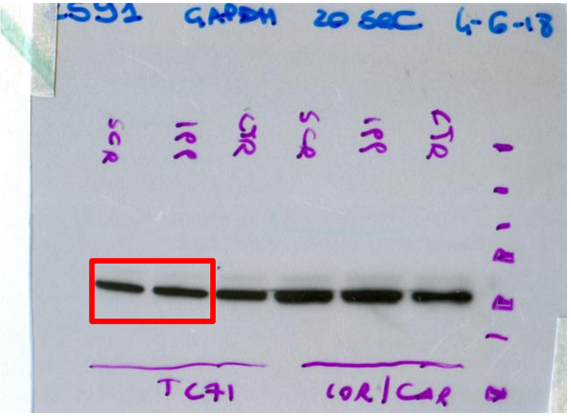

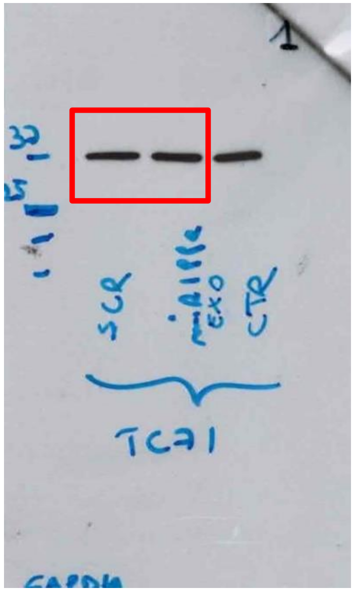

Full unedited gel for **Figure 6**

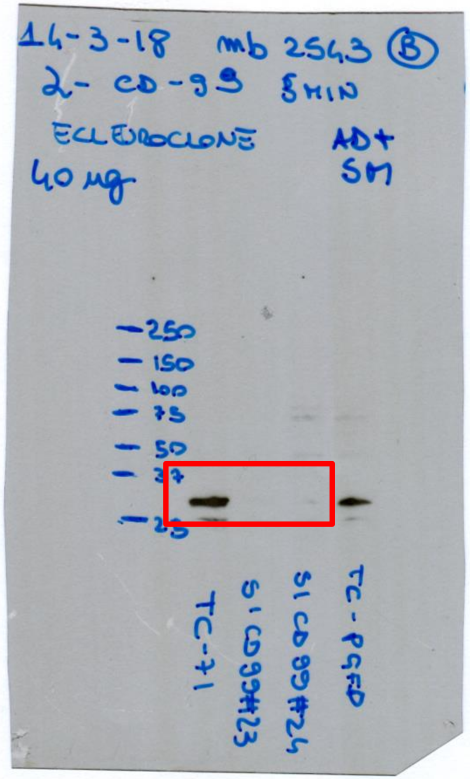

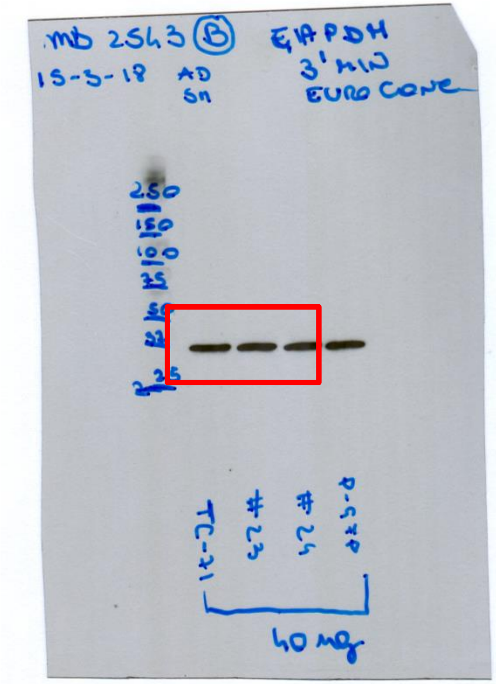

Full unedited gel for **Supplemental Figure 1**

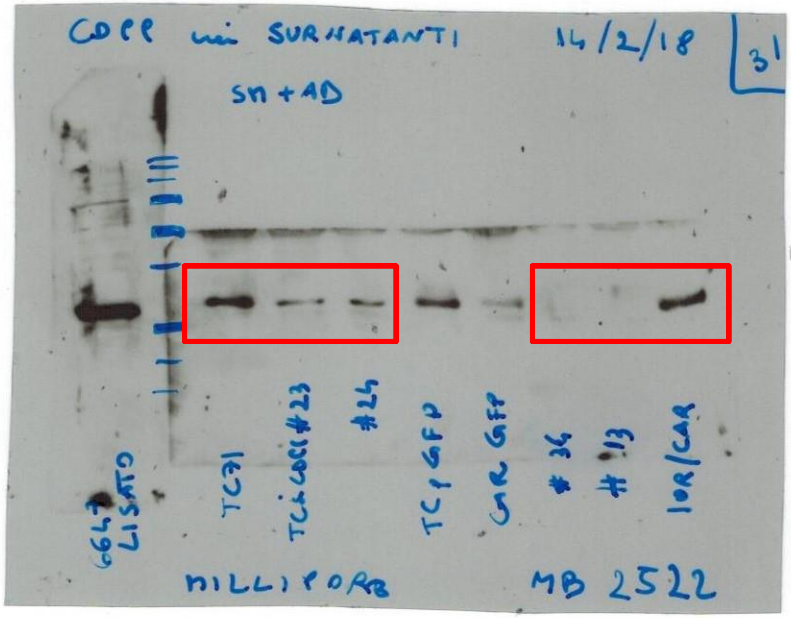

Full unedited gel for **Supplemental Figure 1**

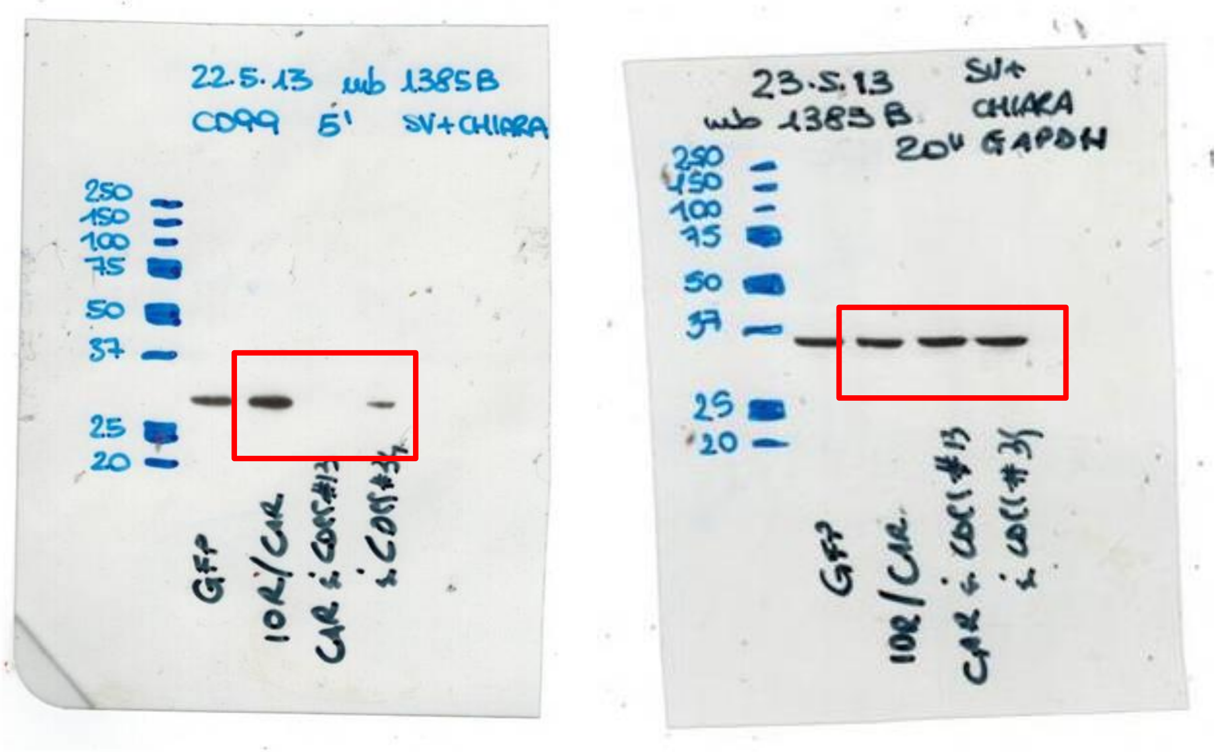

Full unedited gel for **Supplemental Figure 1**

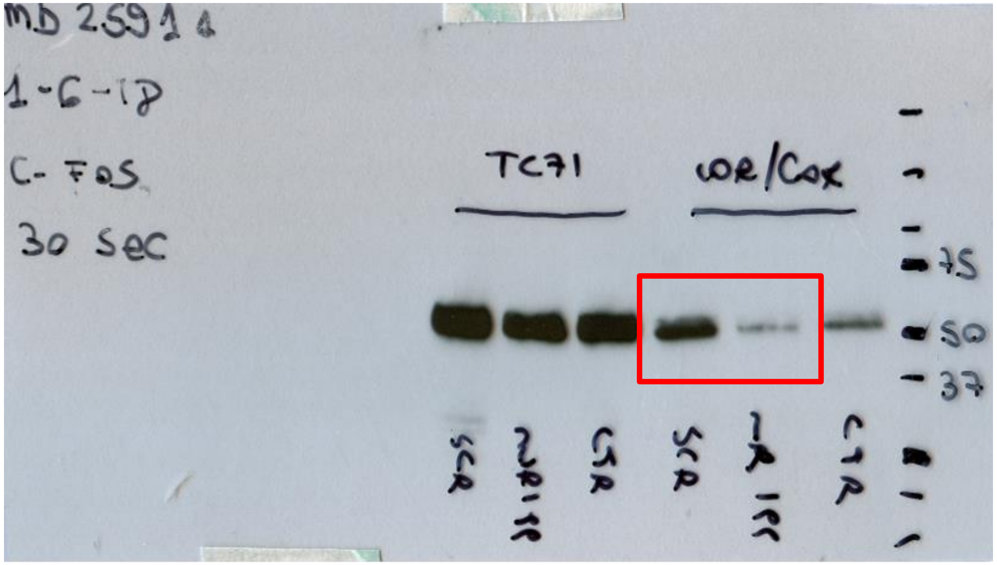

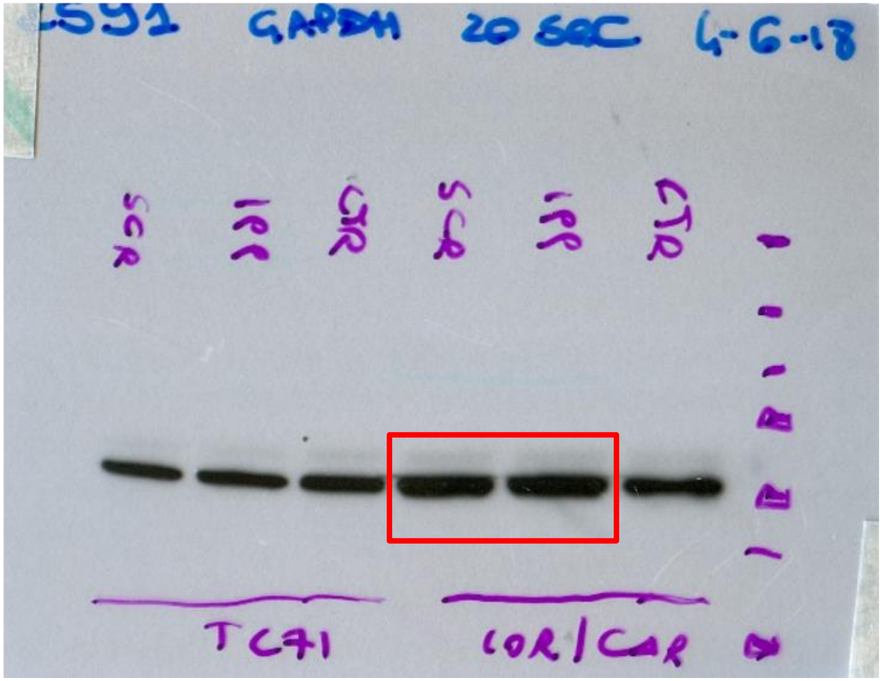

Full unedited gel for **Supplemental Figure 7**
