# Supplementary material for: Exosomes from CD99-deprived Ewing sarcoma cells reverse tumor malignancy by inhibiting cell migration and promoting neural differentiation
Source: Cell Death Dis. 2019 Jun 17;10(7):471. doi: 10.1038/s41419-019-1675-1 (PMC6572819; doi:10.1038/s41419-019-1675-1)
Supplement: Supplementary file 7 — Supplementary figure legends [file 41419_2019_1675_MOESM7_ESM.doc]

**Supplementary Figure 1**

**a** Representative western blot showing CD99 secreted in the supernatants of TC-CD99-shRNA and CAR-CD99-shRNA experimental models. CD99 protein expression from productive cells is shown in comparison. GAPDH was used as a loading control. **b** Size distribution of vesicles derived from EWS cells lines and CD99-silenced cells (TC-71 and TC-CD99-shRNA, IOR/CAR and CAR-CD99-shRNA) analyzed by the Nanosight™ technology. **c** CD99 expression evaluated by flow cytometry on the surface of EXOs from TC-71 and TC-CD99-shRNA cells. The two graphics show in comparison results from vesicles isolated by ExoQuick® or by Ultracentrifuge (*p<0.05, unpaired Student's *t*-test).

**Supplementary Figure 2**

**a** Confocal microscopy image of exosome uptake in untreated TC-71 cells and TC-71 cells treated with CD99neg EXOs. Internalized vesicles, which were labeled with BODIPY® FL C16, were evidenced by a green fluorescent punctuate signal inside the cytoplasm of TC-71 recipient cells. The nuclei were counterstained with Hoechst 33258 (blue). Scale bar: 50 μm. **b** FACS analysis on TC-71 and CD99-silenced cells after exosome exposure. The fluorescent signal confirmed the similar uptake of CD99neg and CD99pos vesicles in recipient cells.

**Supplementary Figure 3**

**a** Immunofluorescence analysis of PI staining in TC-71 and IOR/CAR cell lines receiving or not receiving (CTR) CD99neg EXOs. The cells were counterstained with vital Hoechst 33258 (blue). Scale bar: 100 µm. PI positive cells were counted, and percentages of positive cells are shown on the right panel as the mean ± SE (ns=not significant, Student’s *t*-test). **b** Evaluation of apoptosis by Annexin-V staining in EWS cells treated or not treated (CTR) with CD99neg EXOs.

**Supplementary Figure 4**

**a** Immunofluorescence staining of β-III Tubulin (green) in stable CD99-silenced clones (TC-CD99-shRNA#2 and CAR-CD99-shRNA#1), receiving or not receiving (CTR) CD99pos EXOs derived from their respective parental cell lines. The nuclei were labeled with Hoechst 33258 (blue). Representative images are displayed. Scale bars: 50 µm. **b** Cell migration of TC-CD99-shRNA#2 cells exposed or not exposed (CTR) to CD99pos EXOs. Results of wound-healing assays (left) and motility assays with Transwell chambers (right). The data are shown as the mean ± SE (*p<0.05, Student’s *t*-test).

**Supplementary Figure 5**

Wound-healing was performed in EWS cells, TC-71 and the CD99-silenced variant TC-CD99-shRNA. Each cell line was treated or not (CTR) with both CD99pos or CD99neg EXOs. Scale bars: 100 μm.

**Supplementary Figure 6**

**a** qPCR results showing the relative expression of CD99 in TC-71 cells receiving or not receiving (CTR) CD99neg EXOs for 12h or 24h. The mRNA levels were quantified with the 2^−ΔΔCt^ method. Untreated cells were used as calibrator (2^−ΔΔCt^=1), and GAPDH was used as a housekeeping gene (ns=not significant, Student's *t*-test). **b** Evaluation of CD99 expression by flow cytometry in TC-71 or TC-CD99-shRNA#2 cells treated or not treated (CTR) with CD99neg or CD99pos EXOs for 12h or 24h (ns=not significant, Student's *t*-test).

**Supplementary Figure 7**

**a** Validation of miR-199a-3p expression by qPCR in CD99neg and CD99pos EXOs and their respective producing cells. The relative miRNA levels were normalized to the expression of miR-16 (for EXOs) or RNU6b (for cells). The data are shown as the mean ± SE (*p<0.05, **p<0.01, one-way ANOVA). miR-199a-3p mimic (30 nM) reduced cell growth (trypan blue vital count, **b**) and cell migration (transwell chambers, **c**) in IOR/CAR cells. The data are shown as the mean ± SE of at last three independent experiments (*p<0.05, ***p<0.001, one-way ANOVA). **d** miR-199a-3p mimic (30 nM) decreased c-Fos expression (as evaluated by western blotting) in IOR/CAR cells. SCR, nonspecific control miRNAs. GAPDH was used as a loading control.

**Supplementary Figure 8**

After transfection of miR-199a-3p mimic in TC-71 cells, exosomes enriched with this miRNA were harvested (CD99pos EXOs [miR-199a-3p]) and used to treat CD99-deprived cells, TC-CD99-shRNA. The following parameters were analyzed:

**a** Cell growth by trypan blue count. Columns show the mean values ± SE (*p<0.05, **p<0.01, one-way ANOVA).

**b** Migration by wound-healing assay. Scale bars: 100 µm.

**c** AP-1 transcriptional activity by luciferase assay. Columns represent the mean values ± SE (**p<0.01, Student’s *t*-test). Scrambled (SCR) mimic was used as a transfection control.

**Supplementary Figure 9**

Relative expression of miR-199a-3p in EWS clinical samples as determined by qPCR (mean ± SE). Primary tumors (n=62) and metastases (n=51) were compared. The data were quantified with the 2^-ΔCT^ method. RNU6b was used as a housekeeping gene (*p<0.05, Student’s *t*-test).
